# Neonatal T Follicular Helper Cells Are Lodged in a Pre-T Follicular Helper Stage Favoring Innate Over Adaptive Germinal Center Responses

**DOI:** 10.3389/fimmu.2019.01845

**Published:** 2019-08-13

**Authors:** Beatris Mastelic-Gavillet, Maria Vono, Patrícia Gonzalez-Dias, Frederico Moraes Ferreira, Lucas Cardozo, Paul-Henri Lambert, Helder I. Nakaya, Claire-Anne Siegrist

**Affiliations:** ^1^Departments of Pathology-Immunology and Pediatrics, World Health Organization Collaborating Center for Vaccine Immunology, University of Geneva, Geneva, Switzerland; ^2^Department of Clinical and Toxicological Analyses, School of Pharmaceutical Sciences, University of São Paulo, São Paulo, Brazil; ^3^Laboratory of Immunology, School of Medicine, Heart Institute, University of São Paulo, São Paulo, Brazil

**Keywords:** T follicular helper cells, neonates, vaccines, adjuvant, transcriptional profile analysis

## Abstract

T follicular helper (T_fh_) cells have emerged as a critical limiting factor for controlling the magnitude of neonatal germinal center (GC) reactions and primary vaccine antibody responses. We compared the functional attributes of neonatal and adult T_fh_ cells at the transcriptomic level and demonstrated that the T_fh_ cell program is well-initiated in neonates although the T_fh_ gene-expression pattern (i.e., *CXCR5, IL-21, BCL6, TBK1, STAT4, ASCL2*, and *c-MAF*) is largely underrepresented as compared to adult T_fh_ cells. Importantly, we identified a TH2-bias of neonatal T_fh_ cells, with preferential differentiation toward short-lived pre-T_fh_ effector cells. Remarkably, adjuvantation with CpG-ODNs redirect neonatal pre-T_fh_ cells toward committed GC-T_fh_ cells, as illustrated by increased expression of T_fh_ signature genes and reduced expression of TH2-related genes.

## Introduction

Neonates and young infants share a high vulnerability to infectious diseases. Inducing efficient and sustained B-cell responses remains challenging in this age group (1, 2). Numerous factors concur to limit primary antibody responses, including delayed follicular dendritic cell maturation (3), the limited development and expansion of T follicular helper (T_fh_) cells and, as a result, that of germinal center (GC) B cells and plasma cells (4, 5).

T_fh_ cell differentiation is a multifactorial, multistep process as illustrated by the extensive list of transcription factors [including BCL6 (6–8), ASCL2 (9), LEF-1 (10, 11), TCF-1 (10, 11), BATF (12), STAT3 (13–15), NFAT (16), IRF4 (17), and c-MAF (18)] playing critical and non-redundant roles in driving T_fh_ cell differentiation, from the initiation of their development to their maintenance [reviewed in (19)]. The expression of CXC chemokine receptor 5 (CXCR5) through regulation of KLF2 (20, 21) dictates their spatiotemporal distribution, allowing them to migrate in the B cell zone toward CXCL-13 and entering the B cell follicles (22). Along with CXCR5, co-expression of ICOS (23, 24), PD-1 and IL-21 (25, 26) orchestrates T_fh_ cell differentiation and function. Notably, ICOSL/ICOS signaling plays an important role early in the T_fh_ cell differentiation program by down-regulating negative regulator molecules (20, 23, 27, 28), such as Blimp-1 (29), T-bet (30), and CCR7 (31). Within the follicles, T_fh_ cell development could then be negatively regulated by IL-2 (32) and CTLA-4 (33, 34). Once these checkpoints are crossed, cognate T_fh_-B cell interactions take place for completing T_fh_ cell differentiation.

Over the past years, the specific role of T_fh_ cells as the main providers of B cell help has been unveiled, highlighting a critical role of T_fh_ cells in vaccine elicited immune responses. We previously demonstrated that T_fh_ cell development limits early life GC reactions and resulting primary vaccine antibody responses (4). Notably, adjuvantation of a vaccine with CpG-ODNs was sufficient to partially enhance neonatal antibody responses (4). In this study, we compared the transcriptional profile of neonatal and adult T_fh_ cells and demonstrated that the preferential neonatal polarization toward TH2 is also observed among T_fh_ cells, with increased expression of IL-13 and other TH2-related factors, which may represent an additional negative regulation checkpoint confining activated neonatal CD4^+^ T cells at a pre-T_fh_ stage. Importantly, we showed that adjuvantation with CpG-ODNs reduced the expression of IL-13 and other TH2-related genes and sufficiently strengthened the levels of T_fh_ cell-associated signature molecules to drive the full completion of GC-T_fh_ differentiation.

## Materials and Methods

### Mice

C57BL/6 mice were purchased from Charles River (L'Arbresle, France), bred, and kept in pathogen-free animal facilities in accordance with local guidelines. Mice were used at 1 week (neonates) or 6–8 weeks (adults) of age. All animal experiments were approved by the Geneva Veterinary Office and conducted under relevant Swiss and European guidelines.

### Antigens, Adjuvants, Immunization

Groups of C57BL/6 mice (5–16 mice/group) were immunized intramuscularly with Tetanus Toxoid (TT; 1 limit of flocculation (Lf); gift from Berna Biotech, Bern, Switzerland) adsorbed to aluminum hydroxide [AlOH; Former Novartis Vaccines, Siena, Italy (a GSK Company)] or, when indicated, AlOH plus addition of CpG_1826_ (CpG-ODNs) oligonucleotides (Eurofins MWG Operon). The adult dose of AlOH was weight adjusted to 0.3 mg/adult or 0.15 mg for immunization of 1 week-old mice. The dose of CpG was weight-adjusted as previously described (3) to 50 μg/adult or 3 μg for 1 week-old mice.

### Semiquantitative Real-Time PCR

Total cellular RNA was isolated by RNeasy microkit (Qiagen). cDNA was synthesized from 0.5 μg of total RNA using a mix of random hexamers–oligo d(T) primers and PrimerScript reverse transcriptase enzyme (Takara bio inc. Kit) and pre-amplification was performed with TaqMan® PreAmp Master Mix following supplier's instructions (Applied Biosystems). PCR reactions (10 μl volume) contained diluted cDNA, 2× Power SYBR Green Master Mix (Applied Biosystems), 300 nM of forward and reverse primers. RT-PCRs were performed on a SDS 7900 HT instrument (Applied Biosystems). Each reaction was performed in three replicates, with *EEf1, GusB*, and *MmRPS9* as internal controls genes for data normalization. Raw Ct values obtained with SDS 2.2 (Applied Biosystems) were imported in Excel and normalization factor and fold changes were calculated using the GeNorm method (35). Primer sequences are as follows: *EEf1* sense, 5′-TCCACTTGGTCGCTTTGCT-3′; anti-sense, 5′-CTTCTTGTCCACAGCTTTGATGA-3′, *gusB* sense, 5′-ACGGGATTGTGGTCATCGA-3′; anti-sense, 5′-TGACTCGTTGCCAAAACTCTGA-3′, *MmRPS9* sense, 5′-GACCAGGAGCTAAAGTTGATTGGA-3′; anti-sense, 5′-TCTTGGCCAGGGTAAACTTGA-3′, s1pr2 sense: 5′-TAACTCCCGTGCAGTGGTTTG; anti-sense: 5′-AGAGCGTGATGAAGGCGG-3′, IL-13 sense: 5′-ACAGGACCCAGAGGATATTGCA; antisense: 5′-GGGAGGCTGGAGACCGTAGT-3′, RXRA sense: 5′-AACACAAGTACCCTGAGCAGCC-3′; antisense: 5′-AGGCGGAGCAGCAGCTT-3′, CCR2 sense: 5′-AGAATTGAACTTGAATCATCTGCAA-3′ antisense: 5′-TGTCTTCCATTTCCTTTGATTTGTT-3′, IL7R sense: 5′-AAATGCCCAGGATGGAGACC-3′; antisense: 5′-AAGGAGTGATCGTCCGCGT-3′, and Bcl6 (36), CXCR5 (37), IL-21 (38), Ascl2 (9), c-maf (39), Pou6f1 (40), and PPAR-γ (41) as stated previously.

### Microarray and Analysis

One week-old (16 mice/group) and adult C57BL/6 mice (5 mice/group) were immunized i.m. as described above. Ten days post-vaccination, inguinal draining LNs (dLNs) were pooled per mouse and per group to have a sufficient number of cells. T_fh_ cell populations were isolated by flow-cytometry cell sorting using a MoFlo® Astrios™ flow cytometer (Beckman Coulter). Six independent experiments have been performed to obtain three independent samples per age group. Total RNA was labeled and hybridized on Agilent Whole Mouse Genome Oligo Microarrays 8 × 60 K at Miltenyi Biotec (Germany) and according to the manufacturer's protocol. Arrays were scanned with the Agilent microarray scanner and raw intensities were extracted with Feature Extraction v10.6 software. Raw intensities were integrated, background corrected and log transformed, following the quantile normalization between arrays. Intensities with detection *p*-values <0.01 were arbitrarily discarded. Differentially expressed genes (DEGs) were identified by the ANOVA with Tukey *post-hoc* test considering adjusted *p*-value ≤ 0.05 and fold-change (FC) ≥ 2. Protein-protein interaction networks were built with DEGs using the NetworkAnalyst program (42) and the InnateDB PPIs as database (43). Enrichment analyses were performed with the program Gene Set Enrichment Analysis (GSEA) (44), using customs gene sets of upregulated genes from CD4^+^ T_fh_ effector cells [GSE43863 (45)] and from Bcl6^+^ T_fh_ cells or Bcl6^−^ T_fh_ cells [GSE40068 (8)]. First, raw expression data from GSE43863 and GSE40068 studies were normalized by RMA using the *affy* R/Bioconductor package (46), and submitted to quality control with the *arrayQualityMetrics* R/Bioconductor package (47). For both studies, the up-regulated genes were identified using the R/Bioconductor LIMMA package (48). The T_fh_ effector signature (GSE43863) was generated by comparing CD4^+^ T_fh_ effector cells compared with naïve and TH1 CD4^+^ T cells (adjusted *p*-value < 0.005 and FC ≥ 1.5), while the T_fh_ Bcl6^+^ and Bcl6^−^ signatures (GSE40068) were generated by comparing CD4^+^ CXCR5^+^ Bcl6^+^ and CD4^+^ CXCR5^+^ Bcl6^−^ T cells with CD4^+^ CXCR5^−^ T cells (adjusted *p*-value < 0.05 and FC ≥ 2). Co-expression modules were identified with the *CEMiTool* R/Bioconductor package (49) using variance filter *p*-value < 0.05 and ORA *p*-value < 0.2. CEMiTool package is available at Bioconductor (https://bioconductor.org/packages/release/bioc/html/CEMiTool.html) (49). This package unifies the discovery and the analysis of coexpression gene modules, evaluating whether modules contain genes that are over-represented by specific pathways or that are altered in a specific sample group. Biological and functional enrichment analyses were also performed with the program GSEA using the REACTOME gene sets (50). Finally, unsupervised hierarchical clustering of the samples was carried out via multiscale bootstrap resampling with the PVCLUST R package (51).

## Results

### Transcriptional Profile of Neonatal T_fh_ Cells

**Figure 1:**
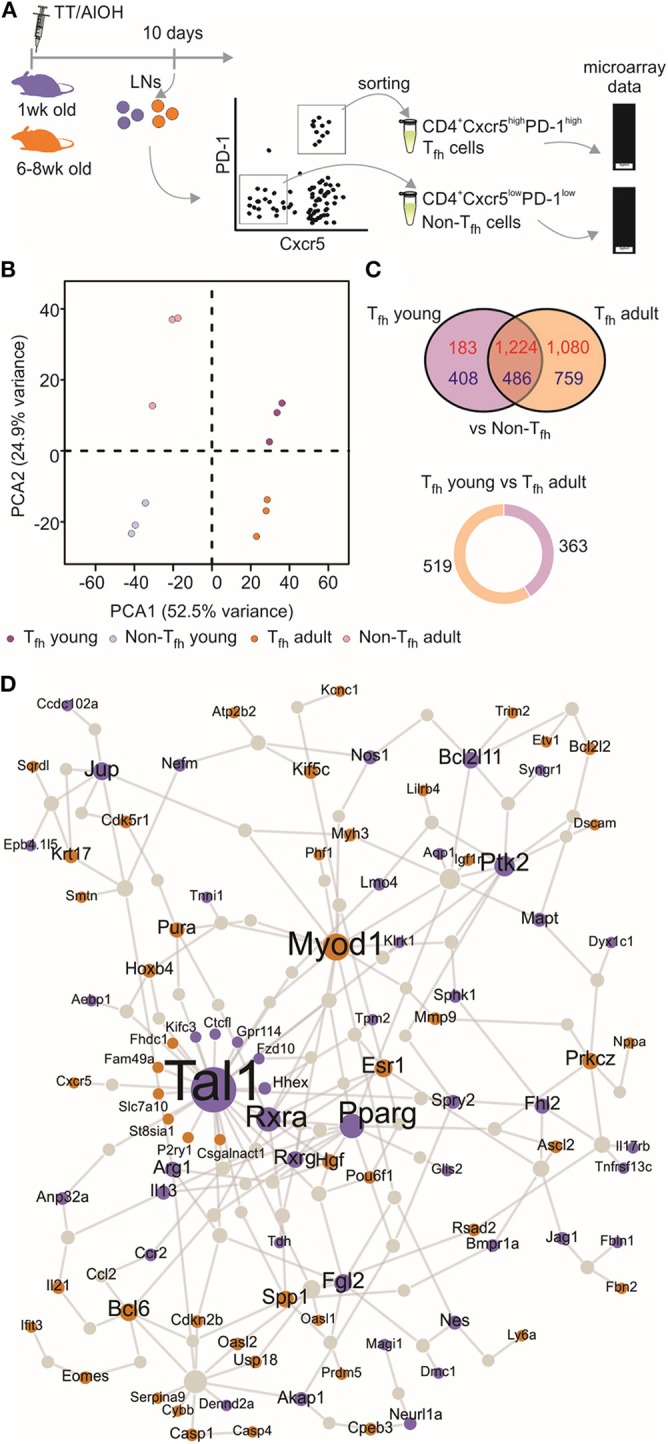
Transcriptional profile of neonatal T_fh_ cells. **(A)** One week-old and adult C57BL/6 mice (5–16 mice/group) were immunized i.m. with TT/AlOH. Ten days post-vaccination the two draining inguinal LNs were collected and pooled to simultaneously isolate CD4^+^ CXCR5^high^PD-1^high^ T_fh_ cells and CD4^+^ CXCR5^−^PD-1^−^ T cells (non-T_fh_) by flow-cytometry cell sorting. The cells obtained from the inguinal draining LNs of either 16 neonates/group or 5 adults/group per experiment were pooled before sorting to recover a sufficient number of cells for experimentation. **(B)** Principal Component Analysis, based on the top 2,000 genes with highest variance, showing (dis)similarities in gene expression across all samples; 1 week (purple, *n* = 3 independent experiments including pools of 16 mice) or adult (brown, *n* = 3 independent experiments including pools of five mice) CD4^+^ CXCR5^high^PD-1^high^ T_fh_ cells and respective controls CD4^+^ CXCR5^−^PD-1^−^ T (non-T_fh_) cells (light colors). **(C)** Venn diagram illustrating the overlap of differentially expressed genes between T_fh_ vs. non-T_fh_ in young and adult immunized mice. Up-regulated genes in T_fh_ cells are shown in red and down-regulated genes in blue. Pie chart shows the proportion of genes differentially up-regulated by 1 week-old T_fh_ cells (purple) when compared to adults, and in brown genes differentially up-regulated by adult T_fh_ cells compared to 1 week-old. **(D)** Protein-protein interaction network constructed with the differentially expressed genes in 1 week-old T_fh_ cells as compared to adults. Up-regulated genes in neonates are illustrated in purple while brown indicates genes up-regulated in adults.

We (4, 52) and others (5) have shown that neonatal T_fh_ cells elicited by aluminum (AlOH)—based adjuvanted vaccines are few and functionally altered compared to adult cells. We therefore investigated the functional attributes of neonatal and adult CD4^+^ CXCR5^high^PD-1^high^ T_fh_ cells at the transcriptomic level. CD4^+^ CXCR5^high^PD-1^high^ T_fh_ and CD4^+^ CXCR5^−^PD-1^−^ T (non-T_fh_) cells were FACS sorted from the draining lymph nodes (LNs) at the previously identified peak (day 10) of the primary germinal center (GC) reaction induced by TT/AlOH (4) for comparative transcription profile analysis (Figure 1A). To visualize the global gene expression patterns of the various subsets, we first performed a principal component analysis (PCA), retaining the top 2,000 genes that contributed most to the total variance (Figure 1B). The projection of the data variance onto the principal components plane efficiently discriminated T_fh_ cells from non-T_fh_ cells in both age groups (Figure 1B), while clustering adult and neonatal T_fh_ cells together. This was confirmed by unsupervised hierarchical analysis, which grouped T_fh_ cells from both age groups (Figure S1). Thus, when successful the T_fh_ differentiation process essentially follows a similar path in early as in adult life.

Nevertheless, the gene expression profiles of neonatal and adult T_fh_ samples differed, revealing functionally differently programmed T_fh_ cells (Figure 1C). Comparing T_fh_ cells from neonatal and adult mice with the corresponding age-matched non-T_fh_ cells identified 2,301 and 3,549 differentially expressed genes, respectively. Overlap comparison showed that 1,710 genes were differentially expressed in T_fh_ cells of both neonatal and adult immunized mice, 591 genes were exclusively differentially expressed in neonatal T_fh_ cells, and 1,839 genes were exclusively differentially expressed in adult T_fh_ cells (Figure 1C).

To get more insight into the key genes leading to functionally differently programmed T_fh_ cells in early or adult life, PPI networks were generated from the differentially up-regulated genes between neonatal and adult T_fh_ cells (Figure 1D). The network derived from the genes differentially expressed in adult T_fh_ cells showed that most of the up-regulated proteins have an established role in T_fh_ biology and function (Bcl6, Ascl2, Pou6f1, IL-21, and Cxcr5). In accordance with the preferential TH2 polarization of early life responses, IL-13 was strongly enriched in neonates vs. adults (Figure 1D). Unexpectedly, three cancer related-pathways genes (Tal1, PPAR-γ, and RXRA) were identified as hub genes in neonates (Figure 1D). Tal1 is expressed early, in hematopoietic stem cells and progenitor cells (53, 54), and subsequently silenced during T-cell development [reviewed in (55)]. It forms a large transcriptional complex with E proteins, LMO family proteins, LDB1, GATA2, and GATA3 (56–58). TAL1, GATA3, and RUNX1 coordinately regulate the expression of downstream target genes. PPAR-γ is a member of the peroxisome proliferator-activated receptor family and forms heterodimer with RXRs to promote their downstream effects, i.e., suppress the transcription of target genes (59). Remarkably, in adults both PPAR-γ (41) and RXRA (60) negatively regulate T cell activation to prevent T_fh_ cell formation. These hub genes may thus play an essential role to functionally alter neonatal T_fh_ cell differentiation.

### T_**fh**_ Cell Differentiation Is Initiated in Neonates but T_**fh**_ Cells Remain Lodged in a pre-T_**fh**_ Stage

**Figure 2:**
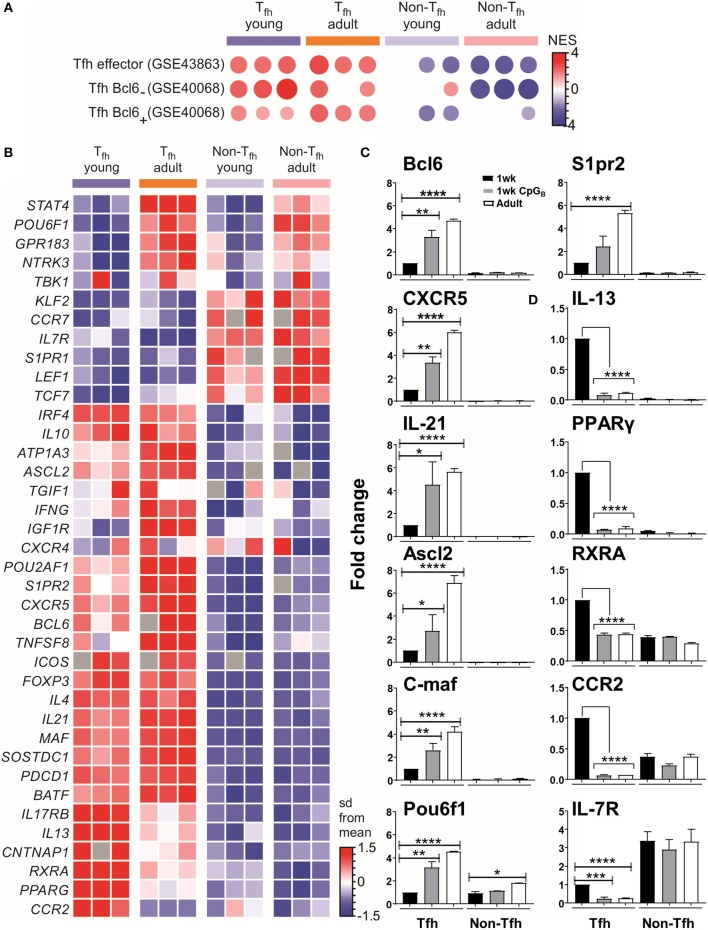
T_fh_ cell differentiation is initiated in neonates but most of the generated cells are lodged in a pre-T_fh_ stage. **(A)** Enrichment analysis of neonatal vs. adult T_fh_ cells with the gene list from T_fh_ effector cells or Bcl6 ^+^ and Bcl6^−^ T_fh_ cells. **(B)** Sample z-score heatmap of significantly differentially expressed genes (row) in T_fh_ cells and their respective controls from young and adult mice (columns). Semi-quantitative RT-PCR analysis of selected T_fh_ cell-related genes **(C)** or TH2 cell-related genes in sorted cells **(D)**, normalized to results obtained for the control genes (EEF1, GusB, RPS9). The graph display mean ± SEM. Cumulative data from adult [TT/AlOH (*n* = 10)] and 1 week-old [TT/AlOH (*n* = 64), TT/AlOH+ CpG_1826_ (*n* = 32)] mice from at least two independent experiments. Statistical analysis was performed with Prism software (Version 7, GraphPad), using unpaired *t*-test. ^*^P < 0.05, ^**^P < 0.01, ^***^P < 0.001, ^****^P < 0.0001.

We then selected the T_fh_ signature genes from published data sets to perform gene set-enrichment analyses (GSEA) with our data. This confirmed that both neonatal and adult cells were enriched for the T_fh_ lineage gene set (GEO accession code GSE43863) (Figure 2A), indicating that the T_fh_ cell differentiation program may succeed in neonates. However, neonatal T_fh_ Bcl6^+^ cells exhibited reduced gene expression signatures compared to adults, while using another GSEA (accession code GSE40068) indicated increased T_fh_ Bcl6^−^ signatures (Figure 2A). Interestingly, Liu et al. demonstrated (8) that CXCR5^+^ Bcl6^low^ cells develop before CXCR5^+^ Bcl6^high^ cells and exhibit a non-polarized gene expression pattern. These “intermediate” T_fh_ cells then further mature into CXCR5^+^Bcl6^high^ T_fh_ cells with the help of cognate B cells (8). This suggests that most neonatal T_fh_ cells are arrested at an early/intermediate stage of T_fh_ development, only a fraction of activated T cells fully up-regulating their expression of key T_fh_ genes, while maintaining their expression of IL-13, one of the preferentially expressed neonatal TH2-related cytokine gene.

To further analyze the transcriptional differences among early life and adult T_fh_ cells, we examined the expression of a set of genes described as up- or down- regulated in T_fh_ cells compared to non-T_fh_ CD4^+^ helper T cells (8, 12, 13, 61, 62). The heatmap of the differentially expressed genes (DEGs) (Figure 2B) confirmed that the gene expression of neonatal and adult T_fh_ samples differed from the control CD4^+^ CXCR5^−^PD-1^−^ samples. Although the overall T_fh_ signature was again present in neonatal T_fh_ cells, the expression levels of *Cxcr5, Batf, c-maf* , and *Il-21* were lower than in adult T_fh_ cells, strengthening our previous observations (4). Compared to adult T_fh_ cells, neonatal T_fh_ cells exhibited much lower expression of Bcl6 (8, 29, 63, 64) and achaete-scute homolog 2 (*Ascl2*) (9), which controls CXCR5 expression and thus the follicular positioning of pre-T_fh_ cells (9). The TBK1 kinase, which controls the maintenance of Bcl6 expression and is thus required for the commitment to the GC-T_fh_ program (27), was also less strongly expressed in neonatal T_fh_ cells.

Bcl6 can bind to promoters and enhancers of genes that encode proteins that control T cell-migration, promoting non-follicular positioning of T cells (7). IL-7R is one of the most repressed T_fh_-relevant genes by Bcl6 and its suppression is critical in T_fh_ cell differentiation (65). Although IL-7R is down-regulated in neonatal T_fh_ cells, its inhibition is almost two times weaker than in adult T_fh_ cells (expression level change of 1.89-fold greater in neonates compared to adults). Liu et al. (65) recently demonstrated that IL-7R expression was inversely correlated with T_fh_ commitment, more precisely with the expression of classical T_fh_ markers: PD-1, CXCR5, and Bcl6. A limited follicular positioning of neonatal pre-T_fh_ cells is also supported by the decreased expression (expression level change of −1.92-fold in neonates compared to adults) of S1pr2, known to suppress CXC12/CXCL13-mediated migration, thus restricting premature egress of T_fh_ cells out of GC (66). Thus, numerous transcription factors contribute to prevent the follicular positioning of neonatal T_fh_ cells, depriving them from interacting with follicular dendritic cells (FDCs) and germinal center B (GC B) cells.

The expression of the *Pou2af1* and *Pou6f1* transcription factors was also reduced. Although their role in T_fh_ cells remains to be fully investigated, *Pou6f1* is expressed in early fate committed T_fh_ cells (6) and *Pou2af1* is highly expressed in early stage GC-T_fh_ cells (10, 67). A recent report by Stauss et al. (68) established that the *Pou2af1* gene promotes Bcl6 expression and T_fh_ cell development. A general reduction in CXCR5 expression was observed on *Pou2af1*^−/−^ CD4^+^ T cells as well as fewer GL7^+^ T_fh_ cells in *Pou2af1*^−/−^ mice (68). Therefore, the POU family transcription factors seems to fine-tune T_fh_ cell development and their reduced expression in neonates may contribute to the limited expression of CXCR5 and GL7 in neonatal T_fh_ cells (4).

The lower expression of STAT4 (expression level change of −2.22-fold in neonates compared to adults) in neonates cements that the specific early life environment prevents the differentiation of pre-T_fh_ cells toward committed GC-T_fh_ cells: the IL-12-STAT4 pathway indeed contributes to the expression of key T_fh_-associated molecules, such as IL-21, CXCR5, and ICOS as well as multiple important transcription factors involved in T_fh_-cell generation, such as Bcl6, c-Maf, and Batf (69, 70).

How does the TH2-like preferential polarization of neonatal effector T cells persist in T_fh_ cells?

Although, TH2 signature genes, including GATA3, IL-4, and IL-5, were not differentially expressed in neonatal T_fh_ cells (Table S1), we observed significant changes in IL-13 and PPAR-γ. Nobs et al. recently showed that PPAR-γ expression in T cells controls the development of type-2 immunity (71). Therefore, the increased expression of PPAR-γ and of additional genes associated with TH2 polarization, including RXRA, ccr2 (72), il17rb (73–75), and cntnap1 (75), may all play a role in maintaining the default TH2-bias of neonatal T_fh_ cells.

Semiquantitative RT-PCR analyses confirmed both the reduced transcript abundance of T_fh_-cell-associated signature genes in neonates (black bars) compared to adults (open bars) (Figure 2C and Figure S2), and their preferential bias toward TH2, as shown by the higher levels of IL-13, PPAR-γ, RXRA, and CCR2 (Figure 2D).

### Adjuvantation With CpG_**1826**_ Bypasses the Neonatal TH2-Bias of pre-T_fh_ Cells and Supports Terminal GC-T_fh_ Cell Differentiation

We and others have shown that administration of TT/AlOH supplemented with TLR9 agonist CpG_1826_ enhanced neonatal antibody responses through the induction of higher T_fh_ and GC B cell numbers (4), as observed in adult mice (76, 77). We thus asked whether neonatal CpG_1826_ adjuvantation induced transcriptional changes in the genes/factors identified as differing between neonatal and adult T_fh_ cells. Semiquantitative RT-PCR on FACS-sorted CD4^+^ CXCR5^high^PD-1^high^ T_fh_ cells isolated 10 days after TT/AlOH + CpG_1826_ immunization showed that CpG adjuvantation increased the transcriptional abundance of T_fh_-cell specific signature genes in neonatal T_fh_ cells (Figure 2C and Figure S2). Flow cytometry analyses confirmed significantly lower CXCR5 expression by neonatal T_fh_ cells (4) (Figure S3A). Remarkably, CpG adjuvantation significantly enhanced the expression of CXCR5 on neonatal T_fh_ cells (Figure S3A), although not to adult like levels. The results support a follicular positioning of neonatal T_fh_ cells, facilitating the T_fh_-GC B cell crosstalk required to provide B cell help during the GC reaction. In contrast, the expression of PD-1 was significantly decreased in 1 week-old and adult mice immunized with CpG-ODNs (Figure S3B).

Notably, TH2-related genes (i.e., PPAR-γ, RXRA, IL-13, and CCR2) were significantly reduced in neonatal T_fh_ cells, reaching similarly low levels as in adult mice immunized without CpG-ODNs (Figure 2D). However, similar to our previous observation (4), IL-4 mRNA transcripts were significantly lower in neonatal Tfh cells and were not affected by CpG adjuvantation (Figure S2B), suggesting that IL-17 transcription would also remain unaffected as previously demonstrated by Debock et al. (5).

Thus, CpG adjuvantation may (1) abrogate the regulation of early life T_fh_ cell differentiation exerted by the TH2-related genes PPAR-γ and RXRA, (2) facilitate the follicular positioning of neonatal T_fh_ cells, as mirrored by increased levels of S1pr2 and CXCR5 and by reduced IL-7R, and (3) support neonatal T_fh_ cell differentiation toward committed GC-T_fh_ cells. This explains our previous observations demonstrating the increase of T_fh_ cell numbers, of GL7 expression by neonatal T_fh_ cells, of GC reactions and thus of Ab titers following neonatal CpG-ODNs adjuvantation (4) (Figures S3C,D).

### Neonatal T_fh_ Cells Preferentially Give Rise to Short-Lived Effector Cells

**Figure 3:**
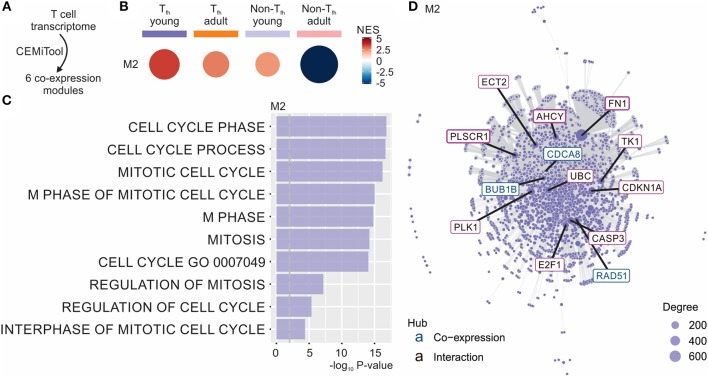
CEMiTool identified co-expression modules in our T_fh_ dataset. **(A)** CEMiTool was run on our T_fh_ dataset and identified 6 co-expression modules. **(B)** Gene Set Enrichment Analyses showing the M2 module activity on each class of samples. NES, normalized enrichment score. The size and color of the circle represents the normalized enrichment score (NES). **(C)** Over Representation Analysis of modules M2 using gene sets from the Reactome Pathway database. Bar graph shows the –log_10_ adjusted P-value of the enrichment between genes in modules and gene sets from Reactome Pathway database. The pathways were ordered by significance as indicated in the x-axis. The vertical dashed gray line indicates an adjusted *P*-value of 0.01. **(D)** Interaction plot for M2, with gene nodes highlighted. The nodes represent the 413 genes of M2 plus the genes added by protein-protein interaction information. The genes are connected by co-expression and/or protein-protein interaction. Gene network of module M2 for the most connected genes (hubs) are labeled and colored based on their “origin”: if originally present in the CEMiTool module, they are colored blue; if inserted from the interactions, they are colored red. The size of the node is proportional to its degree.

To better understand the fate of the pre-T_fh_ cells elicited in early life, we next ran the Co-Expression Molecules identification Tool (CEMiTool) (49) on our data set. CEMiTool is an R package that provides in an automated manner unsupervised gene filtering, automated parameter selection for identifying modules, enrichment and module functional analyses as well as integration with interactome data (49). This modular expression analysis identified 6 different co-expression modules (Figure 3A and Data Sheet S1), of which only module M2 was significantly enriched in neonates (Figure 3B). This module was enriched for genes related to cell cycle (49) (Figure 3C), suggesting the capacity of neonatal T_fh_ cells to enter the cell cycle more rapidly than their adult counterparts. CEMITool also integrates co-expression analysis with protein-protein interaction data. Expression of important genes associated with cell-cycle progression, including gene encoding E2F1 and TK1, were identified as hubs in module M2 (Figure 3D). This early life characteristic was previously observed (78–80): neonatal T and B lymphocytes have the capacity to enter the cell cycle more quickly and thus efficiently mobilize responses from an otherwise completely naive population—possibly to compensate for the limitations in immune cell function in early life (i.e., lack of immunological memory) (78–80). Yet, rapid cycle entry only gives rise to short-lived effector cells (78–80).

**Figure 4:**
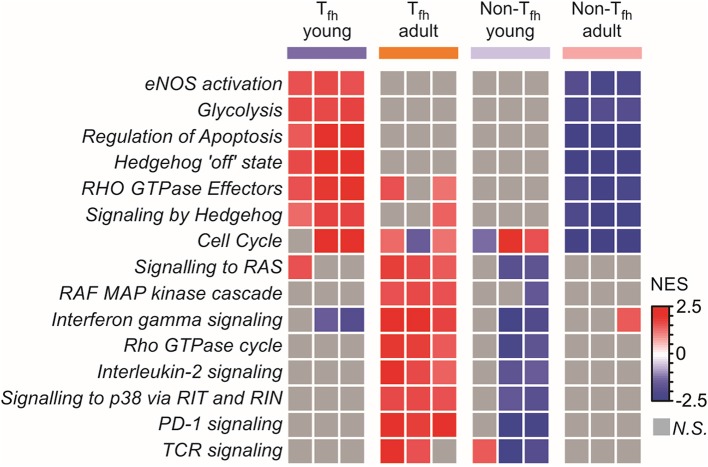
Pathway analysis reveals cell intrinsic differences between neonatal and adult T_fh_ cells. Gene Set enrichment analysis using Reactome gene sets. Heat map showing the normalized enrichment scores for the pathways consistently induced or repressed in a T_fh_ young or adult population are illustrated by color.

To complete our observations, we performed a pathway analysis which revealed cell-intrinsic differences between neonatal and adult T_fh_ cells (Figure 4): neonatal T_fh_ cells were enriched in pathways associated with cell proliferation, apoptosis and key metabolic reactions, such as glycolysis, considered to play an important role in T cell activation and differentiation, while adult cells were enriched in mitogen-activated protein kinase (MAPK)-signaling pathways, thus outlining age-associated differences in the maturity and basic function. Interestingly, enrichment of Hedgehog signaling, which predispose T cell differentiation toward the TH2 pathway (81), further supports the TH2-bias of neonatal T_fh_ cells. Altogether, these transcriptional analyses of neonatal vs. adult T_fh_ cells reveal the existence of multiple coordinated regulatory mechanisms resulting into the preferential differentiation of neonatal CD4^+^ T cells toward innate, short-lived pre-T_fh_ effectors rather than adaptative (GC-derived) immunity defense mechanisms.

## Discussion

We previously identified the induction of T_fh_ cells as limiting early life GC and Ab responses elicited by vaccines including aluminum-based adjuvants (4). We now demonstrate that the few T_fh_ cells elicited in early life retain a preferential bias toward TH2, strongly expressing IL-13, and PPAR-γ and RXRA which negatively regulate T_fh_ cell differentiation (41, 60), and that numerous transcription factors contribute to restrict activated neonatal CD4^+^ T cells at a pre-T_fh_ cell stage of short-lived effectors favoring innate rather than GC-associated adaptive responses. Importantly, we show that this fate is not inevitable as adjuvantation with CpG-ODNs reduced the expression of TH2-related genes and sufficiently strengthened the T_fh_ cell-associated signature molecules to drive the GC-T_fh_ differentiation program to its completion and fine tune the GC reaction.

Following immune challenges, neonatal responses are often weak (82). This has been associated with a propensity of neonatal T cells to give rise to short-lived effector cells (78–80) and to produce elevated levels of TH2-type cytokines compared to adults (83–85). We show that these two key neonatal characteristics persist during T_fh_ cell differentiation, lodging the cells in a pre-T_fh_ stage characterized by a TH2 bias. Our results suggest that a delicate balance of several signals known to promote TH2 development may contribute in maintaining an optimal environment for the TH2-biased T_fh_ cell differentiation in neonates, including increased expression of PPAR-γ (71, 86), RXRA (87), ccr2 (72), il17rb (73–75), cntnap1 (75), Hedgehog signaling (81), and lower levels of c-maf mRNA transcripts. A critical role for c-maf in limiting TH2 responses and in driving T_fh_ cell development was recently unveiled by Andris et al. (18). Further investigations are warranted to delineate whether Tal1 may also play a fundamental role in the generation and persistence of TH2-biased T_fh_ cells in neonates.

A limitation of our study is that the very few T_fh_ cells induced in early life (about 2 × 10^4^ T_fh_ cells from a pool of 8 neonates) and thus the small amount of recovered RNA precluded the analysis of all potentially interesting genes. As the microarray did not reveal significant changes in TH17-related genes, in GATA3 or in IL-5 expression in neonatal T_fh_ vs. non-T_fh_ cells (Table S1), we did not compare their mRNA transcript levels to those of adult cells. In our model, both IFN-γ and IL-4 mRNA transcript levels are significantly lower in neonatal T_fh_ cells—and not affected by CpG adjuvantation (Figure S2B). The similar expression of Foxp3 in neonatal and adult T_fh_ cells was confirmed by semiquantitative RT-PCR (Figure S2D). Altogether, these results suggest that in our model, neonatal T_fh_ cells do not exhibit a bias toward TH1, TH17 nor Treg cells. Our attempts to develop validated assays to reliably measure several proteins in the few recovered neonatal T_fh_ cells did not succeed, and such proteins remained below detection levels when assessed in lymph nodes homogenates (not shown).

PPAR-γ and RXRA were identified as critical hub genes in neonates. Therefore, besides their role in maintaining the overall TH2 bias, PPAR-γ and RXRA may also negatively regulate T_fh_ cell differentiation. PPAR-γ is known to (71, 86) promote Tregs survival (88–90) and inhibit the formation of T_fh_ cells and GC reactions via the regulation of Bcl6 and IL-21 (41). That inhibition of Bcl6 expression is illustrated in neonatal T_fh_ cells by increased expression of the TH2-related gene, IL-13, previously identified as one of the most repressed Bcl6-target gene (65). A role for Tgif1-RXR interaction in the establishment or inhibition of a chronically elevated T_fh_ cell population was recently computationally predicted and demonstrated by Leber et al. (60); an increase with Tgif1 was associated with an increase in the T_fh_ response, while an increase in RXR was more closely correlated with the T_fh_ decline phase (60). Small changes in RXRA, such as 10% change in expression were previously demonstrated to result in a 50% change in activity and significant alteration of downstream transcriptional targets (91). We conclude that the differential expression of TH2-related genes PPAR-γ and RXRA might be involved in the distinct genetic programming of neonatal and adult T_fh_ cells.

Although the T_fh_ cell program is well-initiated in neonates, the gene-expression pattern of neonatal T_fh_ cells underrepresented that of adult T_fh_ cells, suggesting that T-B interactions fail to elicit appropriate signals and provide efficient help to neonatal pre-T_fh_ cells to further differentiate into committed GC-T_fh_ cells. Indeed, T_fh_ cells differentiation involves a multi-signal process that includes expression of CXCR5, IL-21, Bcl6, TBK1, STAT4, Ascl2, and c-maf which were all expressed to lower levels as compared to adult T_fh_ cells. Remarkably, adjuvantation with CpG-ODNs, skewed neonatal pre-T_fh_ cells toward committed GC-T_fh_ cells, as illustrated by increased expression of T_fh_-signature genes (Figure 2C and Figure S2). In parallel, genes associated with follicular positioning of T_fh_ cells were increased (i.e., s1pr2, Ascl2, and CXCR5), facilitating the cognate T_fh_-B cell interactions for completing T_fh_ cell differentiation (92–96), with concomitant increase in Bcl6 and IL-21 expression (10, 95). Ascl2 directly regulates the localization of T_fh_ cells via CXCR5 expression and suppression of CCR7 and PSGL1 (9). CXCR5 allows T_fh_ cells to migrate into the B cell follicles and form stable contacts with antigen-primed B cells (92, 97). These results indicate that a combination of several T_fh_-specific signals, in addition to previously described environment factors and CD4^+^ T cells intrinsic determinants (4), maintain a favorable environment for TH2-biased T_fh_ cell differentiation, restricting neonatal CD4^+^ T cells at a pre-T_fh_ stage of short-lived effector cells. Adjuvantation with CpG-ODNs is sufficient to counteract the TH2-biased response of neonatal T_fh_ cells, reducing TH2-related genes to adult-like levels, while T_fh_ signature genes (i.e., Bcl6, CXCR5, IL-21, Ascl2, C-maf, Pou6f1, s1pr2, Batf, CXCR4, and TBK1) are progressively enhanced, resulting in differentiated and GC-committed T_fh_ cells. As illustrated in Figures 2C,D, the switch from pre-T_fh_ to mature T_fh_ cells involves changes in the expression levels of several factors—such that “classical” mechanistic approaches including knockout/knock-in mice were not attempted.

We recently demonstrated that adjuvantation of a vaccine with a liposome including a C-type lectin receptor agonist was able to elicit potent GC reactions in neonates after a single dose (98). Altogether, these results show that immune deficiencies seen in early life can be overcame by providing the right signals, and are in accordance with the current understanding that the neonatal immune system is not deficient but tightly regulated to best adapt to the unique challenge of a rapidly required adaptation from a sterile to a microbial environment (99).

Further studies are necessary to investigate whether abrogating the TH2 bias of T_fh_ cells in early life is critical for the full commitment of T_fh_ cell differentiation and the subsequent GC B cell and antibody responses, resulting in effective responses to vaccination in early life.

## Data Availability

The data has been deposited at the Gene Expression Omnibus repository—accession number is GSE126843.

## Ethics Statement

This study was carried out in accordance with the recommendations of the Geneva Veterinary Office and conducted under relevant Swiss and European guidelines. The protocol was approved by the Geneva Veterinary Office.

## Author Contributions

BM-G, P-HL, and C-AS contributed to formulation of theory and prediction. BM-G, P-HL, and C-AS designed the research. BM-G and MV performed the experiments and analyzed and/or interpreted the data. BM-G, MV, and C-AS wrote the manuscript. PG-D, FF, LC, and HN performed the microarray analysis and critically revised the manuscript. All authors reviewed the manuscript.

### Conflict of Interest Statement

The authors declare that the research was conducted in the absence of any commercial or financial relationships that could be construed as a potential conflict of interest.

## Supplementary Material

Figure S1 Unsupervised hierarchical analysis groups together T_fh_ cells from both age groups. Hierarchical clustering of CD4^+^ CXCR5^high^PD-1^high^ T_fh_ cells and respective controls CD4^+^ CXCR5^−^PD-1^−^ T cell samples was obtained by pvclust R package. CD4^+^ CXCR5^high^PD-1^high^ T_fh_ cells from young (purple) and adult (orange) mice and their respective controls (light colors) formed distinct groups.

Figure S2 CpG adjuvantation is sufficient to increase Bcl6 targeted T_fh_ specific genes, such as Atp1a3 as well as T_fh_ cell-related genes, including BATF, TBK1, and CXCR4. One week-old and adult C57BL/6 mice (5–8 mice/group) were immunized i.m. with TT/AlOH. Ten days post-vaccination the draining LNs were collected to simultaneously isolate CD4^+^ CXCR5^high^PD-1^high^ T_fh_ cells and CD4^+^ CXCR5^−^PD-1^−^ T (non-T_fh_) cells by flow-cytometry cell sorting. The cells obtained from the two inguinal draining LNs of either 16 neonates/group or 5 adults/group per experiment were pooled before sorting to recover a sufficient number of cells for experimentation. Semi-quantitative RT-PCR analysis of selected T_fh_ cell-related genes **(A)**, IL-4 **(B)**, TH1 cell-related genes **(C)**, Foxp3 **(D)**, or Bcl6 targeted T_fh_ specific genes **(E)** in sorted cells, normalized to results obtained for the control genes (EEF1, GusB, RPS9). The graph display mean ± SEM. Cumulative data from adult [TT/AlOH (*n* = 10)] and 1 week-old [TT/AlOH (*n* = 64), TT/AlOH+ CpG_1826_ (*n* = 32)] mice from at least two independent experiments. Fold changes are shown relative to 1 week-old mice immunized with TT/AlOH. Statistical analysis was performed with Prism software (Version 7, GraphPad), using unpaired *t*-test. ^*^*P* < 0.05, ^**^*P* < 0.01, ^***^*P* < 0.001, ^****^*P* < 0.0001.

Figure S3 Progressive increase of TT-specific IgG1 titers and T_fh_ cell responses in neonates with CpG adjuvantation. C57BL/6 mice were immunized i.m. at 1 week or as adult with TT/AlOH with or without CpG (5–8 mice per group). The adult dose of AlOH was weight adjusted to 0.3 mg/adult or 0.15 mg for immunization of 1 week-old mice. **(A)** CXCR5 and **(B)** PD-1 surface marker mean fluorescence intensity (MFI) on T_fh_ cells from 1 week and adult mice day 10 post-immunization. TT-specific **(C)** IgG1 or **(D)** IgG2a titers 10 days post-immunization. Numbers are represented as mean ± SEM. Data are representative of one of three independent experiments. Mann–Whitney *U* test differences with *p* > 0.05 were considered to be insignificant. ^*^*p*-value (0.01–0.05), ^**^*p*-value (0.001–0.01), ^***^*p*-value (<0.001).

Table S1 Summary table of TH1, TH2, TH17, and Treg selected genes. Indicated are the *p*-values, Median intensity values of all samples, the flag counts and the ProbeID. Fold-change values (columns C–E) which did not pass the selection criteria (Anova *P*-value ≤ 0.05, Tukey *P*-value ≤ 0.05, fold-change ≥2 or ≤-2, and reliable detection of the signal (Flag counts) ≤1 in the group with higher expression) are in black color, while up-regulated ones are shown in red and down-regulated ones are shown in green.

Data Sheet s1 CEMiTool output html file for all modules.
