# Supplementary figures and images for: Neonatal T Follicular Helper Cells Are Lodged in a Pre-T Follicular Helper Stage Favoring Innate Over Adaptive Germinal Center Responses
Source: Front Immunol. 2019 Aug 13;10:1845. doi: 10.3389/fimmu.2019.01845 (PMC6700230; doi:10.3389/fimmu.2019.01845)

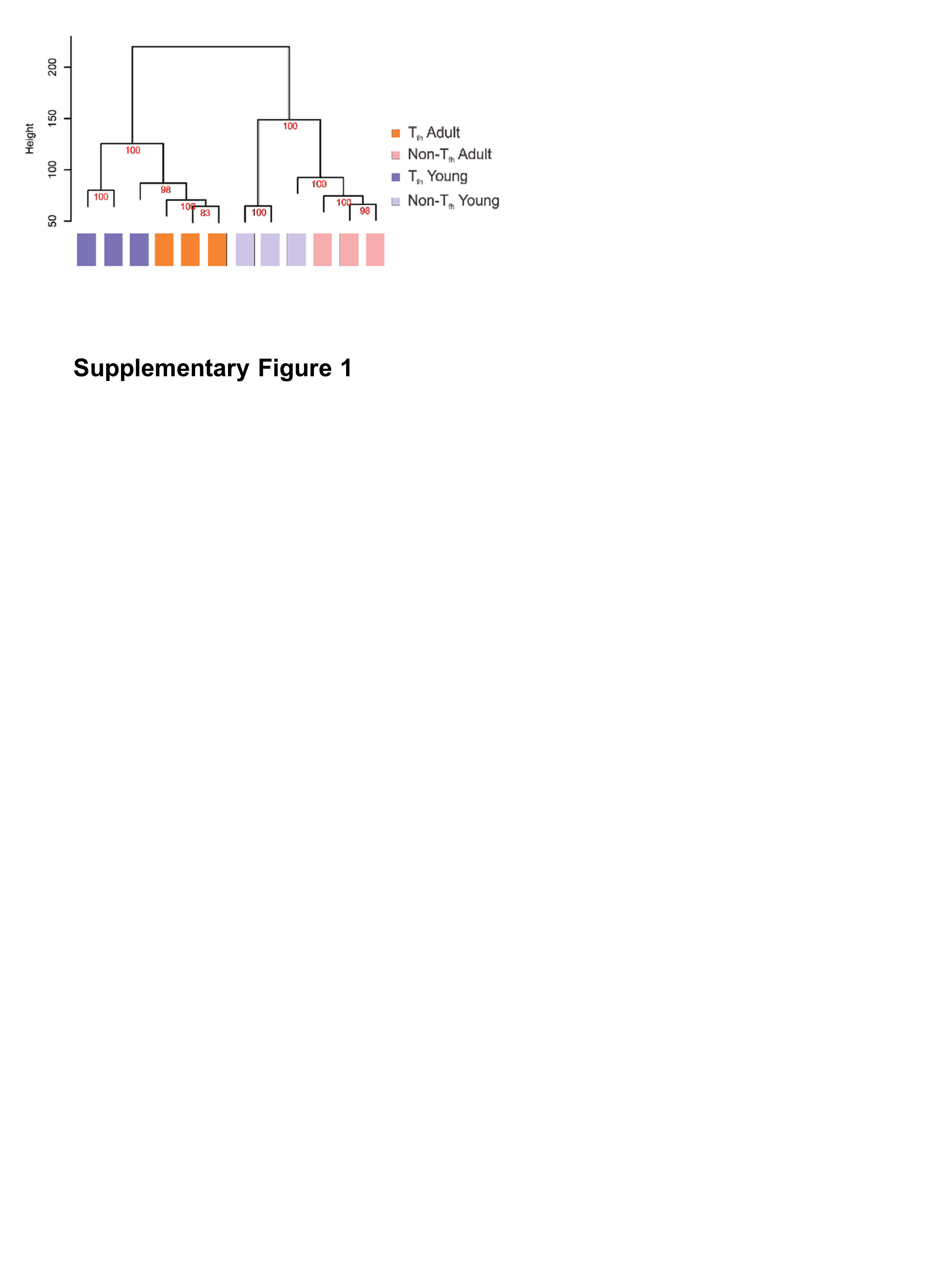

Supplement: Figure S1 — Unsupervised hierarchical analysis groups together Tfh cells from both age groups. Hierarchical clustering of CD4+ CXCR5highPD-1high Tfh cells and respective controls CD4+ CXCR5−PD-1− T cell samples was obtained by pvclust R package. CD4+ CXCR5highPD-1high Tfh cells from young (purple) and adult (orange) mice and their respective controls (light colors) formed distinct groups. [file Image_1.TIF]

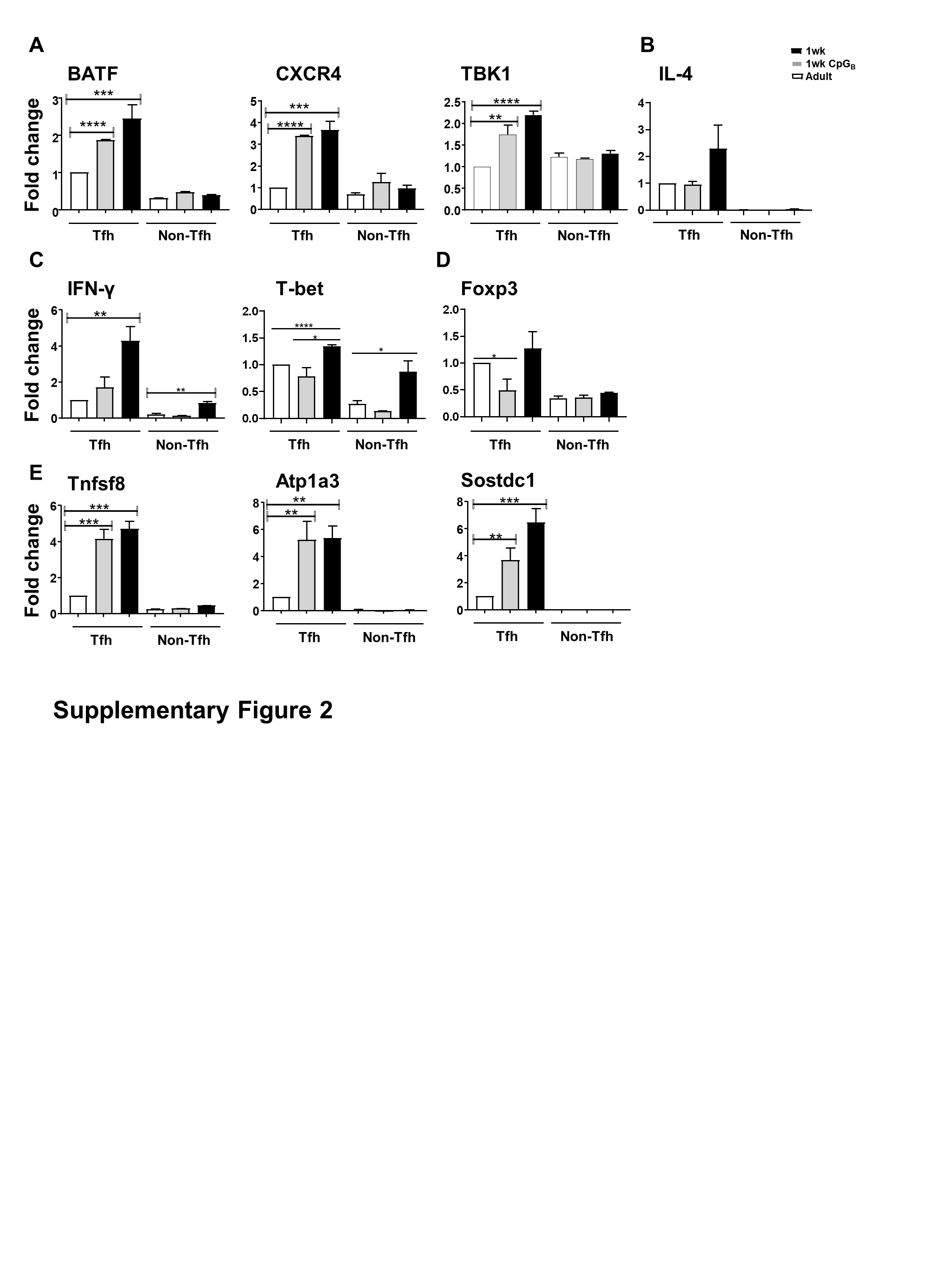

Supplement: Figure S2 — CpG adjuvantation is sufficient to increase Bcl6 targeted Tfh specific genes, such as Atp1a3 as well as Tfh cell-related genes, including BATF, TBK1, and CXCR4. One week-old and adult C57BL/6 mice (5–8 mice/group) were immunized i.m. with TT/AlOH. Ten days post-vaccination the draining LNs were collected to simultaneously isolate CD4+ CXCR5highPD-1high Tfh cells and CD4+ CXCR5−PD-1− T (non-Tfh) cells by flow-cytometry cell sorting. The cells obtained from the two inguinal draining LNs of either 16 neonates/group or 5 adults/group per experiment were pooled before sorting to recover a sufficient number of cells for experimentation. Semi-quantitative RT-PCR analysis of selected Tfh cell-related genes (A), IL-4 (B), TH1 cell-related genes (C), Foxp3 (D), or Bcl6 targeted Tfh specific genes (E) in sorted cells, normalized to results obtained for the control genes (EEF1, GusB, RPS9). The graph display mean ± SEM. Cumulative data from adult [TT/AlOH (n = 10)] and 1 week-old [TT/AlOH (n = 64), TT/AlOH+ CpG1826 (n = 32)] mice from at least two independent experiments. Fold changes are shown relative to 1 week-old mice immunized with TT/AlOH. Statistical analysis was performed with Prism software (Version 7, GraphPad), using unpaired t-test. *P < 0.05, **P < 0.01, ***P < 0.001, ****P < 0.0001. [file Image_2.TIF]

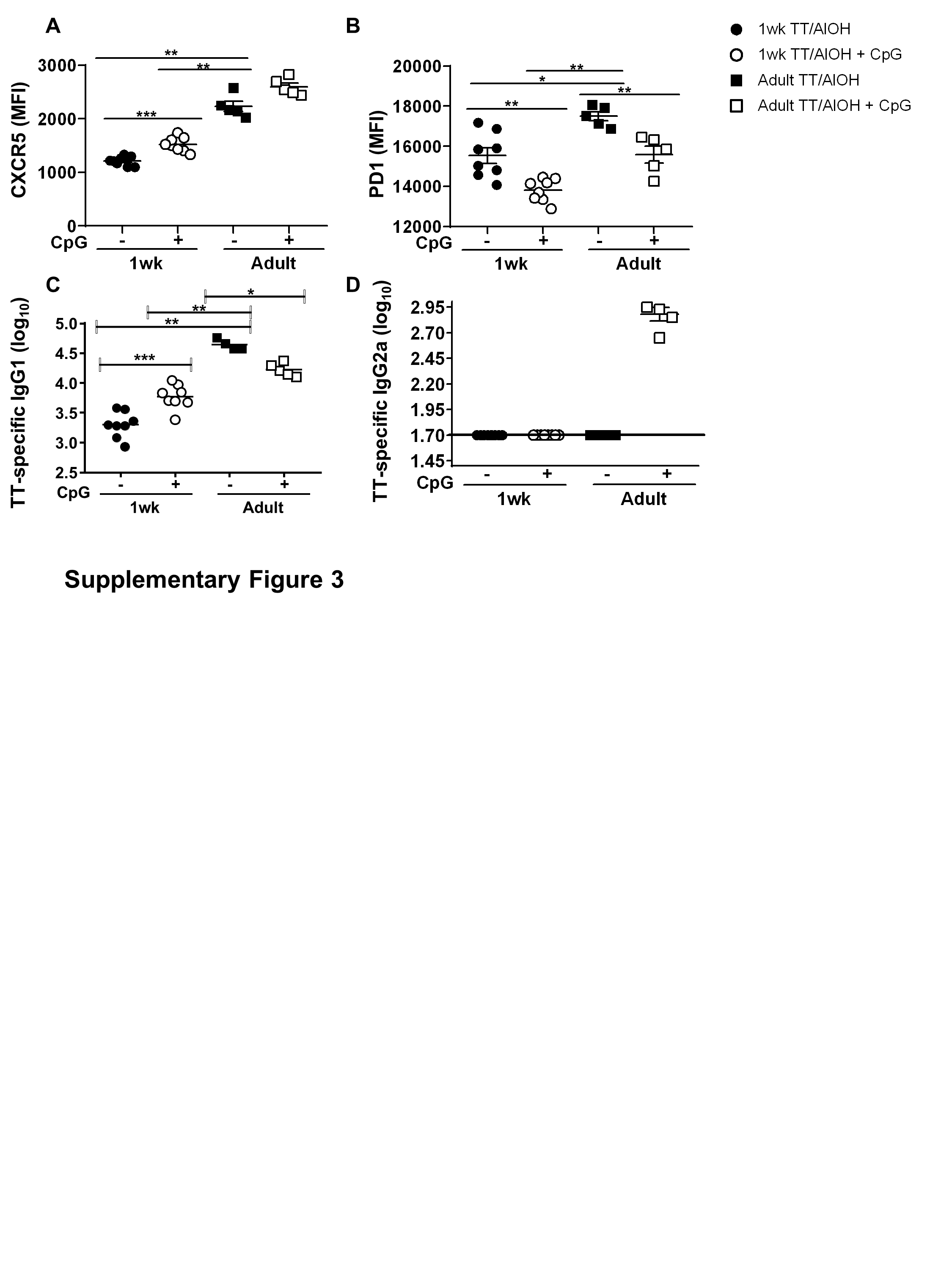

Supplement: Figure S3 — Progressive increase of TT-specific IgG1 titers and Tfh cell responses in neonates with CpG adjuvantation. C57BL/6 mice were immunized i.m. at 1 week or as adult with TT/AlOH with or without CpG (5–8 mice per group). The adult dose of AlOH was weight adjusted to 0.3 mg/adult or 0.15 mg for immunization of 1 week-old mice. (A) CXCR5 and (B) PD-1 surface marker mean fluorescence intensity (MFI) on Tfh cells from 1 week and adult mice day 10 post-immunization. TT-specific (C) IgG1 or (D) IgG2a titers 10 days post-immunization. Numbers are represented as mean ± SEM. Data are representative of one of three independent experiments. Mann–Whitney U test differences with p > 0.05 were considered to be insignificant. *p-value (0.01–0.05), **p-value (0.001–0.01), ***p-value (<0.001). [file Image_3.TIF]
