# Supplementary material for: Neonatal T Follicular Helper Cells Are Lodged in a Pre-T Follicular Helper Stage Favoring Innate Over Adaptive Germinal Center Responses
Source: Front Immunol. 2019 Aug 13;10:1845. doi: 10.3389/fimmu.2019.01845 (PMC6700230; doi:10.3389/fimmu.2019.01845)
Supplement: Table S1 — Summary table of TH1, TH2, TH17, and Treg selected genes. Indicated are the p-values, Median intensity values of all samples, the flag counts and the ProbeID. Fold-change values (columns C–E) which did not pass the selection criteria (Anova P-value ≤ 0.05, Tukey P-value ≤ 0.05, fold-change ≥2 or ≤-2, and reliable detection of the signal (Flag counts) ≤1 in the group with higher expression) are in black color, while up-regulated ones are shown in red and down-regulated ones are shown in green. [file Table_1.pdf]

	TFH 1 wk vs TFH adult	TFH 1wk vs non TFH 1 wk	TFH 1 adult vs non TFH adult	Anova P		TFH 1 wk vs TFH adult	TFH 1wk vs non TFH 1 wk	TFH 1 adult vs non TFH adult			Flags based on detection p values			
GeneName	Fold_Change	Fold_Change	Fold_Change	P-Value	Adj.p-value	Tukey.p-vlaue	Tukey.p-vlaue	Tukey.p-vlaue	Median	ProbeName	TFH_1wk	TFH_adult	non_TFH_1w	non_TFH_adult
Eomes	-2.379	3.436	4.687	7.241E-06	3.432E-04	3.211E-03	3.023E-04	5.991E-05	8.667	A_55_P2150717	0	0	0	0
Fasl	-1.349	-1.373	-1.537	3.728E-02	1.213E-01	4.707E-01	4.270E-01	2.089E-01	9.014	A_55_P2016114	0	0	0	0
Ifng	-2.575	3.725	4.359	2.537E-03	1.803E-02	1.473E-01	3.872E-02	2.215E-02	10.929	A_52_P68893	0	0	0	0
Ifngr1	1.074	-1.563	-1.562	8.935E-04	8.645E-03	8.624E-01	5.257E-03	5.290E-03	12.685	A_51_P391445	0	0	0	0
Il12rb2	1.178	-1.226	-1.613	5.203E-01	6.701E-01	9.586E-01	9.255E-01	5.138E-01	2.358	A_55_P1978416	3	3	3	2
Il18rap	2.017	3.428	1.912	1.660E-04	2.727E-03	1.368E-02	3.910E-04	2.090E-02	6.164	A_52_P517098	0	0	0	0
Il2ra	1.554	3.055	2.130	1.429E-05	5.235E-04	1.866E-02	4.205E-05	6.841E-04	11.964	A_55_P1980796	0	0	0	0
Stat1	-1.480	-1.167	-1.339	5.366E-03	3.107E-02	8.247E-02	6.863E-01	2.262E-01	7.225	A_55_P1955906	0	0	0	0
Tbx21	1.170	4.089	1.792	9.435E-04	8.966E-03	8.954E-01	1.111E-03	1.188E-01	10.312	A_51_P501364	0	0	0	0
Ccr1	-1.051	1.003	1.044	2.191E-02	8.294E-02	3.627E-02	9.979E-01	7.088E-02	1.885	A_52_P616356	3	3	3	3
Gata3	-1.257	1.737	1.757	1.412E-03	1.187E-02	3.568E-01	1.189E-02	1.061E-02	12.121	A_66_P111011	0	0	0	0
Il13	6.709	37.757	8.326	4.506E-05	1.116E-03	8.654E-03	1.214E-04	4.571E-03	4.314	A_55_P2180839	0	0	2	3
Il1rl1	1.255	1.045	1.870	1.233E-01	2.853E-01	7.291E-01	9.968E-01	2.693E-01	9.445	A_55_P2027737	0	0	0	2
Il4	1.002	19.744	12.383	2.820E-07	5.098E-05	1.000E+00	1.295E-06	4.739E-06	9.474	A_51_P237865	0	0	0	0
Il5	1.036	1.041	1.087	9.890E-01	9.911E-01	9.996E-01	9.995E-01	9.952E-01	2.433	A_55_P2138386	2	3	3	3
Pparg	7.014	15.886	4.491	3.739E-06	2.256E-04	2.230E-04	1.702E-05	1.333E-03	5.718	A_51_P106799	0	0	0	0
Ahr	1.374	2.067	1.094	3.278E-01	5.469E-01	8.192E-01	2.673E-01	9.943E-01	2.708	A_65_P06603	1	2	3	3
Il17a	1.616	1.950	2.549	5.106E-01	6.655E-01	9.618E-01	8.772E-01	7.904E-01	4.180	A_55_P2156697	0	1	1	2
Il17f	1.077	-2.339	1.043	4.180E-01	6.232E-01	9.994E-01	5.612E-01	9.999E-01	3.481	A_51_P519301	1	2	1	3
Il17re	1.210	-1.281	-1.866	7.054E-02	1.925E-01	7.964E-01	6.478E-01	6.645E-02	7.133	A_55_P2014427	0	0	0	0
Il21r	-1.475	-1.143	-1.354	3.057E-02	1.050E-01	2.587E-01	8.971E-01	4.441E-01	11.261	A_55_P2071447	0	0	0	0
Rora	1.084	5.733	4.794	2.105E-06	1.607E-04	9.437E-01	1.097E-05	2.472E-05	9.027	A_55_P2078123	0	0	0	0
Rorc	1.309	2.016	1.060	3.979E-02	1.271E-01	5.350E-01	2.732E-02	9.896E-01	7.183	A_55_P2051094	0	0	0	0
Ccl4	-2.220	1.493	3.190	3.347E-01	5.542E-01	6.641E-01	9.339E-01	3.865E-01	8.151	A_51_P509573	0	0	0	0
Ccr6	-1.263	6.410	6.233	9.575E-08	2.819E-05	2.309E-01	7.898E-07	8.938E-07	12.114	A_55_P2108943	0	0	0	0
Fosl1	2.365	1.985	1.396	2.045E-02	7.899E-02	7.380E-02	1.698E-01	6.808E-01	2.559	A_51_P308796	1	3	3	3
Foxp3	1.090	4.252	3.406	3.547E-07	5.829E-05	8.013E-01	1.604E-06	5.670E-06	11.347	A_55_P2032703	0	0	0	0
Ikzf2	-1.090	8.614	10.215	2.861E-07	5.122E-05	9.415E-01	3.144E-06	1.760E-06	10.285	A_66_P122551	0	0	0	0
Il10	1.239	30.372	28.038	3.986E-04	4.928E-03	9.838E-01	2.202E-03	2.562E-03	5.594	A_51_P430766	0	0	2	1
Il1rn	-2.071	1.201	2.074	2.241E-01	4.314E-01	3.776E-01	9.716E-01	3.756E-01	2.046	A_55_P1962400	3	2	3	3
Irf8	-1.073	1.989	3.929	2.638E-03	1.854E-02	9.929E-01	1.185E-01	3.839E-03	11.170	A_52_P354823	0	0	0	0
Myb	-1.005	1.047	2.587	1.745E-02	7.054E-02	1.000E+00	9.980E-01	2.912E-02	12.075	A_55_P2017826	0	0	0	0
Nr4a1	1.160	1.436	1.490	6.371E-01	7.297E-01	9.858E-01	8.438E-01	8.032E-01	14.830	A_51_P239654	0	0	0	0
Nr4a3	1.384	2.096	1.812	6.211E-01	7.202E-01	9.702E-01	7.561E-01	8.527E-01	5.337	A_51_P128397	0	0	0	0
Tgif2	1.047	-1.230	-1.151	1.897E-01	3.851E-01	9.747E-01	3.159E-01	6.081E-01	6.920	A_66_P121285	0	0	0	0
Tnfsf11	-1.385	6.157	10.926	3.124E-06	2.041E-04	4.099E-01	7.516E-05	9.714E-06	8.833	A_55_P2039359	0	0	0	0
