# Supplementary material for: Neonatal T Follicular Helper Cells Are Lodged in a Pre-T Follicular Helper Stage Favoring Innate Over Adaptive Germinal Center Responses
Source: Front Immunol. 2019 Aug 13;10:1845. doi: 10.3389/fimmu.2019.01845 (PMC6700230; doi:10.3389/fimmu.2019.01845)
Supplement: Data Sheet s1 — CEMiTool output html file for all modules. [file Data_Sheet_1.pdf]

CEMiTool

Contents

1 Report **1**
- 1.1 Modules . . . 1
- 1.2 Profile Plot . . . 1
- 1.3 Gene Set Enrichment Analysis . . . 4
- 1.4 Over Representation Analysis . . . 5
- 1.5 Interaction Network . . . 11
- 1.6 Parameters . . . 13

1 Report

1.1 Modules

1.2 Profile Plot

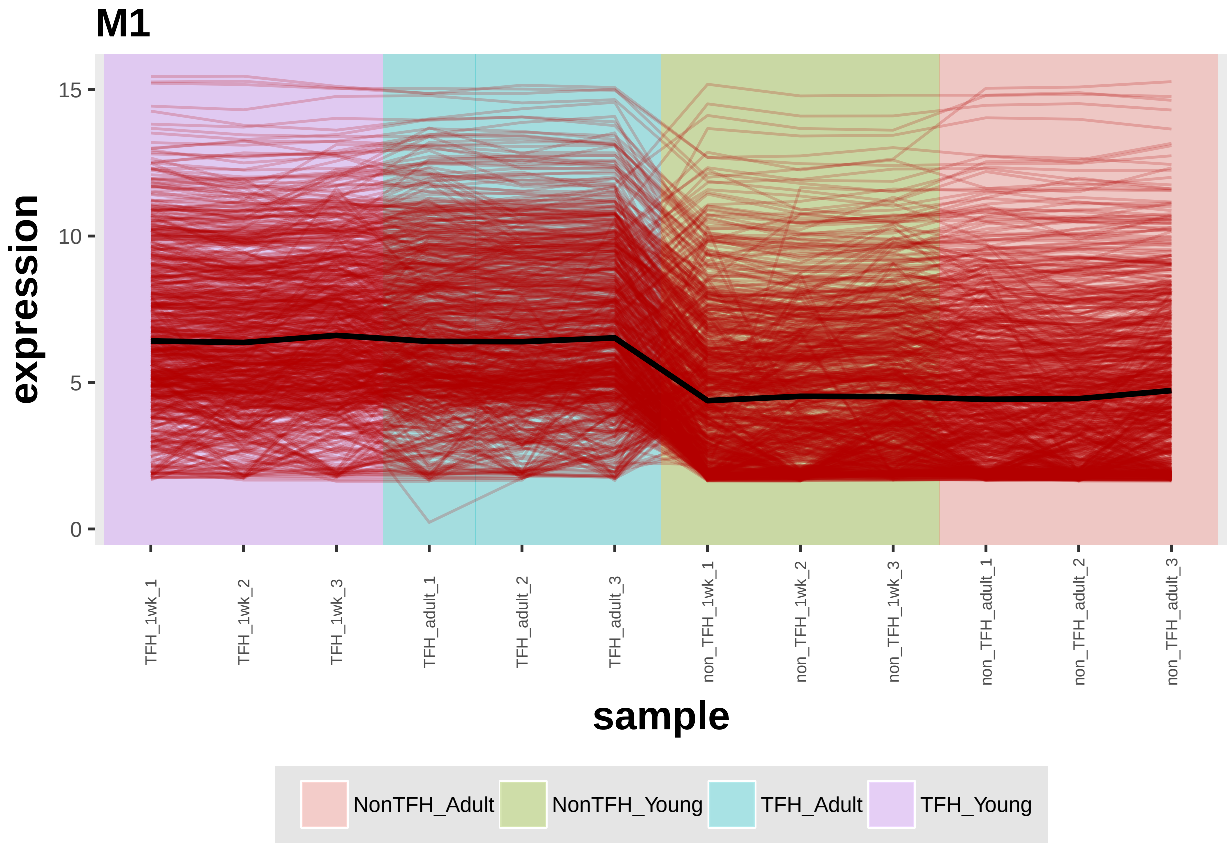

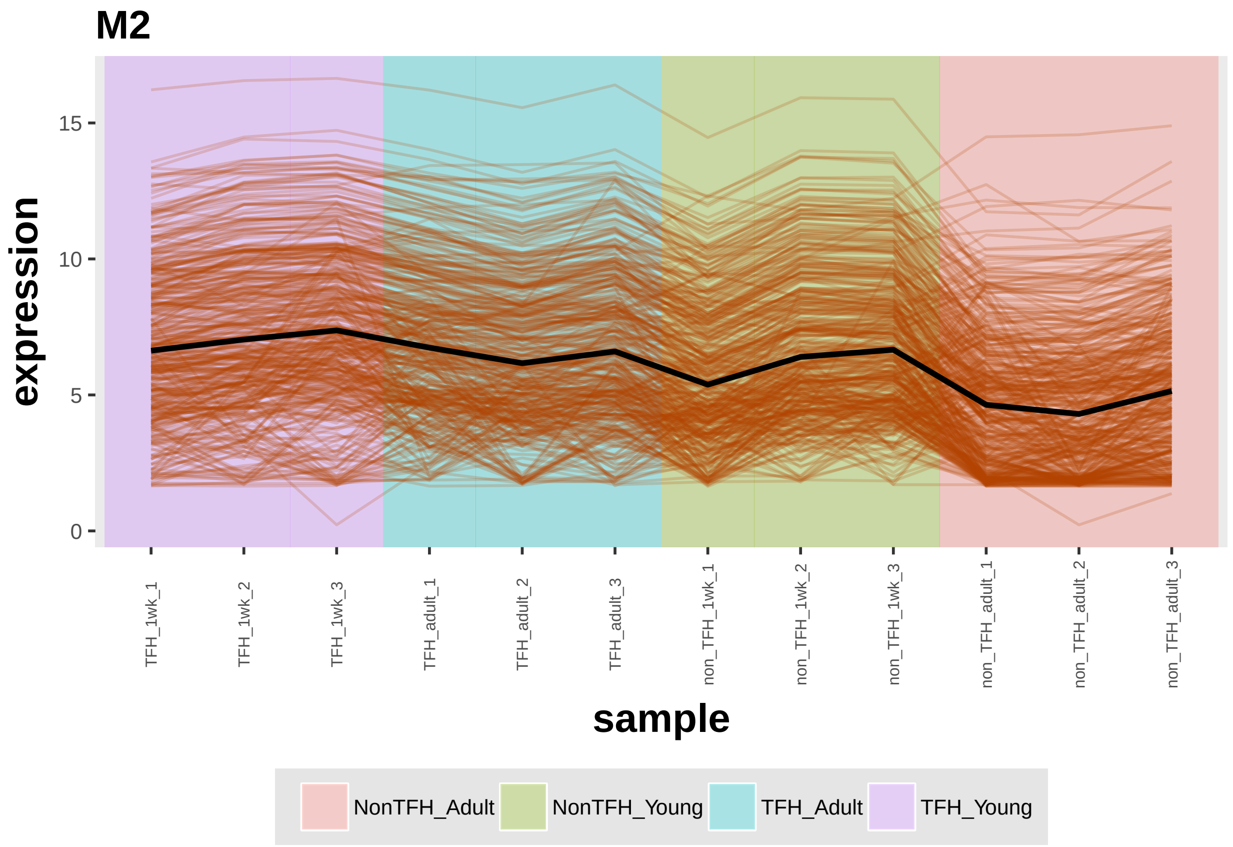
M2
expression
15
10
5
0
TFH_1wk_1
TFH_1wk_2
TFH_1wk_3
TFH_adult_1
TFH_adult_2
TFH_adult_3
non_TFH_1wk_1
non_TFH_1wk_2
non_TFH_1wk_3
non_TFH_adult_1
non_TFH_adult_2
non_TFH_adult_3
sample
NonTFH_Adult
NonTFH_Young
TFH_Adult
TFH_Young

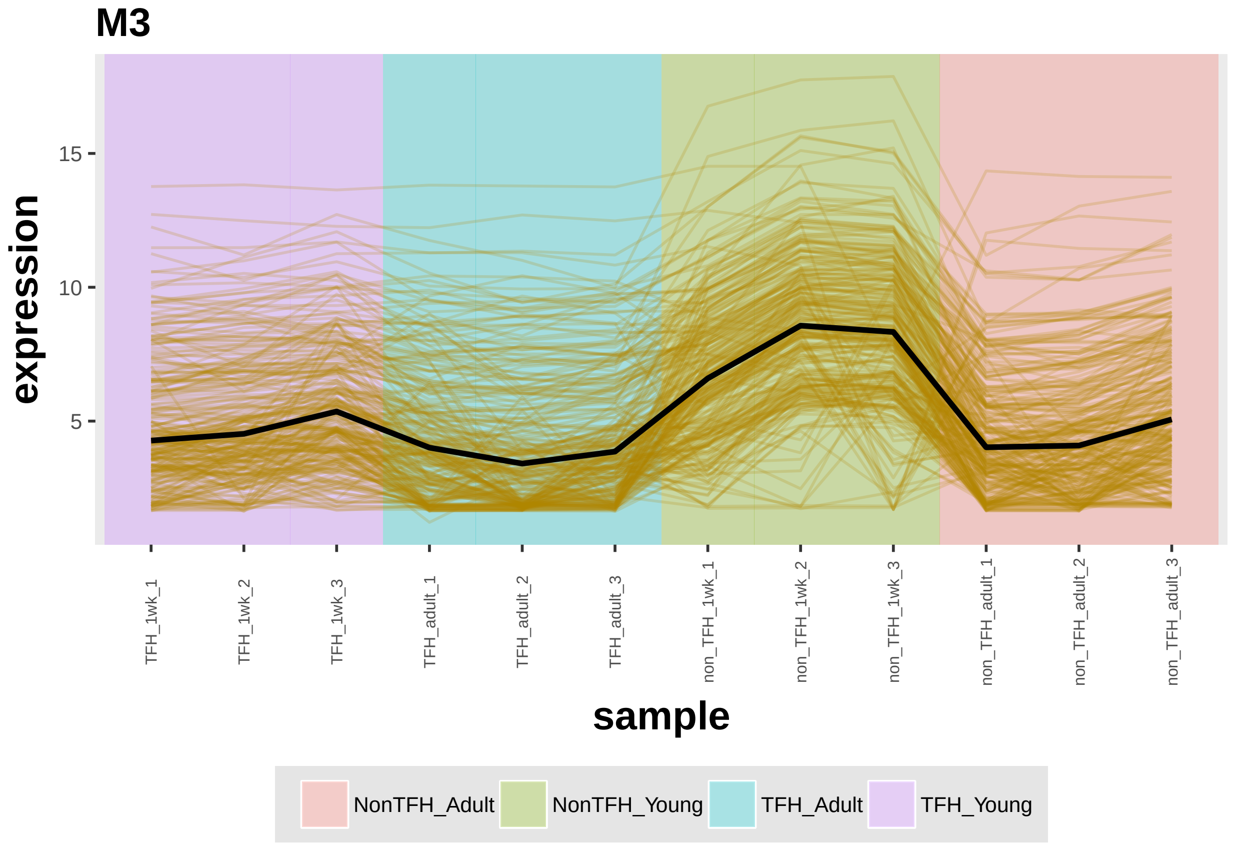
M3
expression
15
10
5
TFH_1wk_1
TFH_1wk_2
TFH_1wk_3
TFH_adult_1
TFH_adult_2
TFH_adult_3
non_TFH_1wk_1
non_TFH_1wk_2
non_TFH_1wk_3
non_TFH_adult_1
non_TFH_adult_2
non_TFH_adult_3
sample
NonTFH_Adult
NonTFH_Young
TFH_Adult
TFH_Young

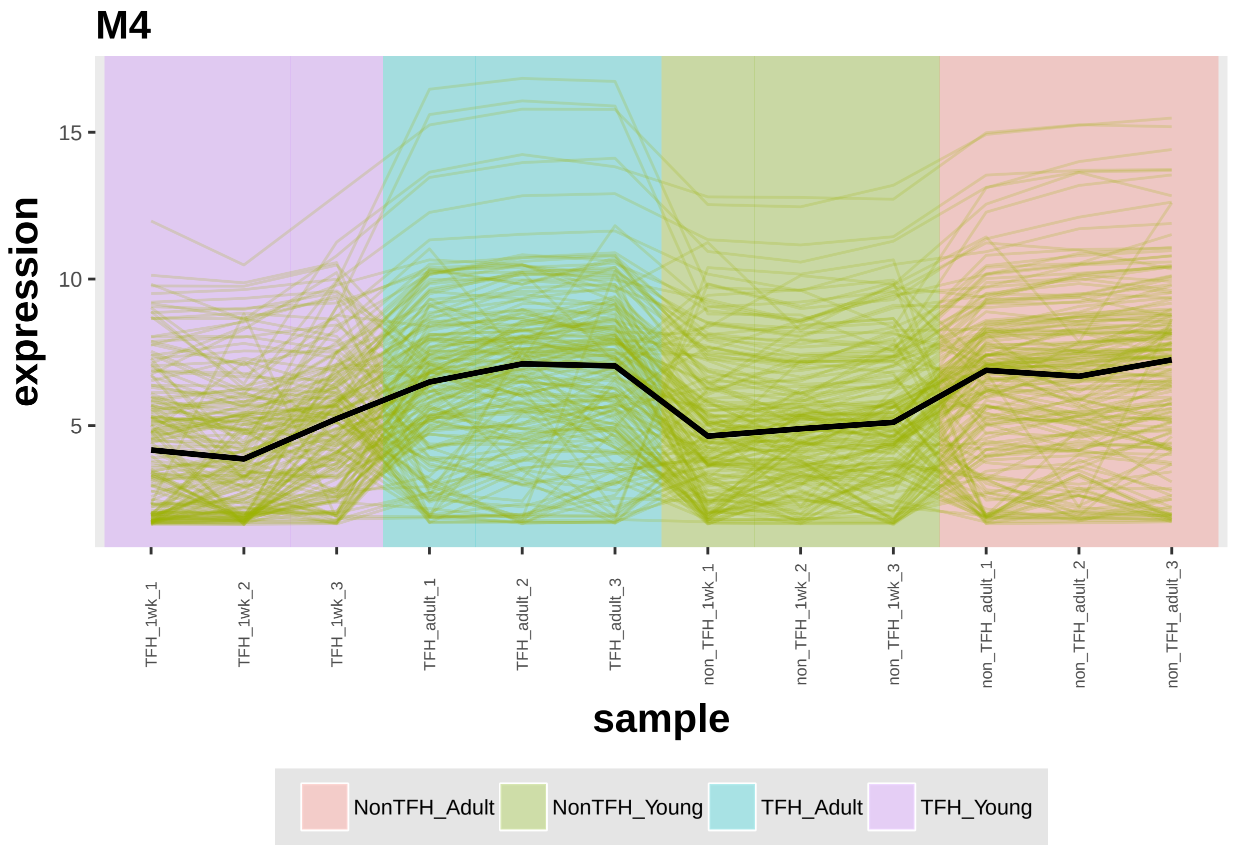
M4
expression
15
10
5
TFH_1wk_1
TFH_1wk_2
TFH_1wk_3
TFH_adult_1
TFH_adult_2
TFH_adult_3
non_TFH_1wk_1
non_TFH_1wk_2
non_TFH_1wk_3
non_TFH_adult_1
non_TFH_adult_2
non_TFH_adult_3
sample
NonTFH_Adult
NonTFH_Young
TFH_Adult
TFH_Young

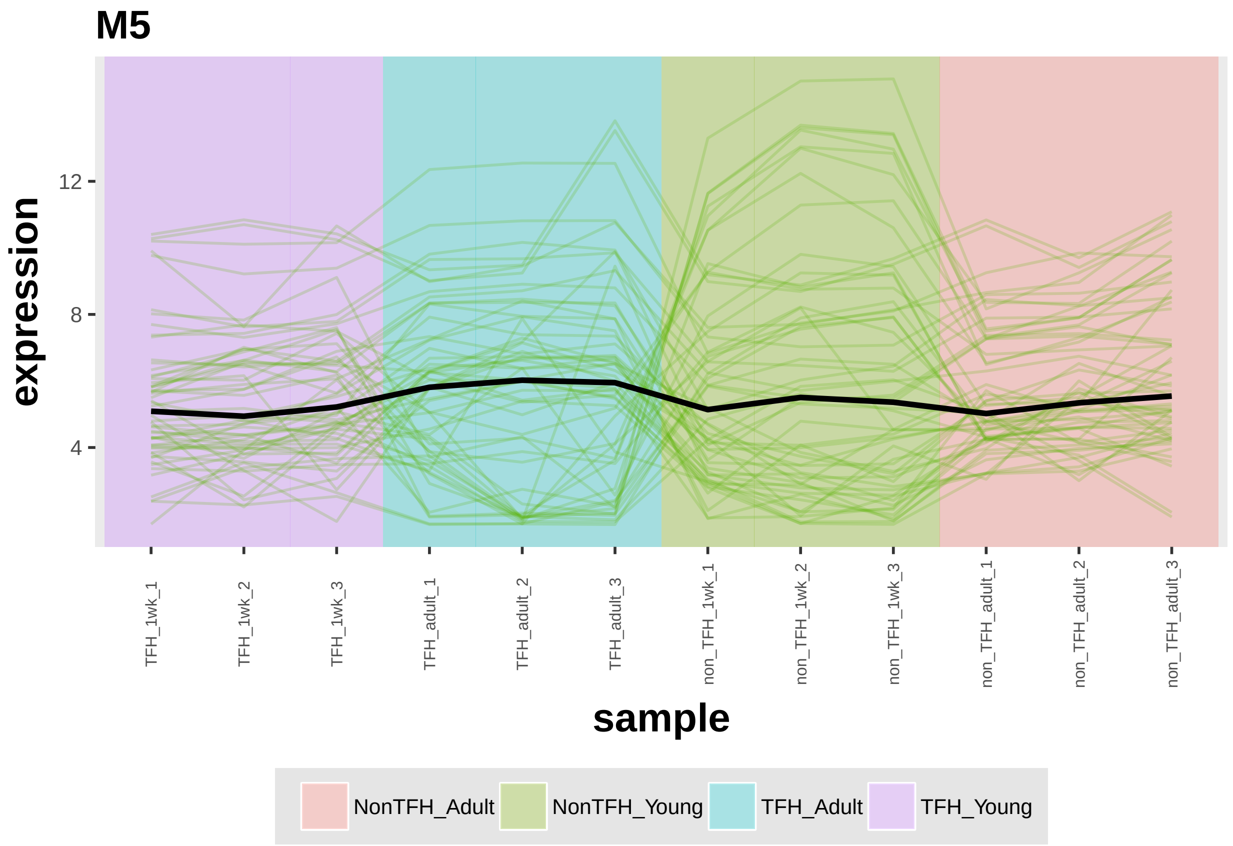
M5
expression
12
8
4
TFH_1wk_1
TFH_1wk_2
TFH_1wk_3
TFH_adult_1
TFH_adult_2
TFH_adult_3
non_TFH_1wk_1
non_TFH_1wk_2
non_TFH_1wk_3
non_TFH_adult_1
non_TFH_adult_2
non_TFH_adult_3
sample
NonTFH_Adult
NonTFH_Young
TFH_Adult
TFH_Young

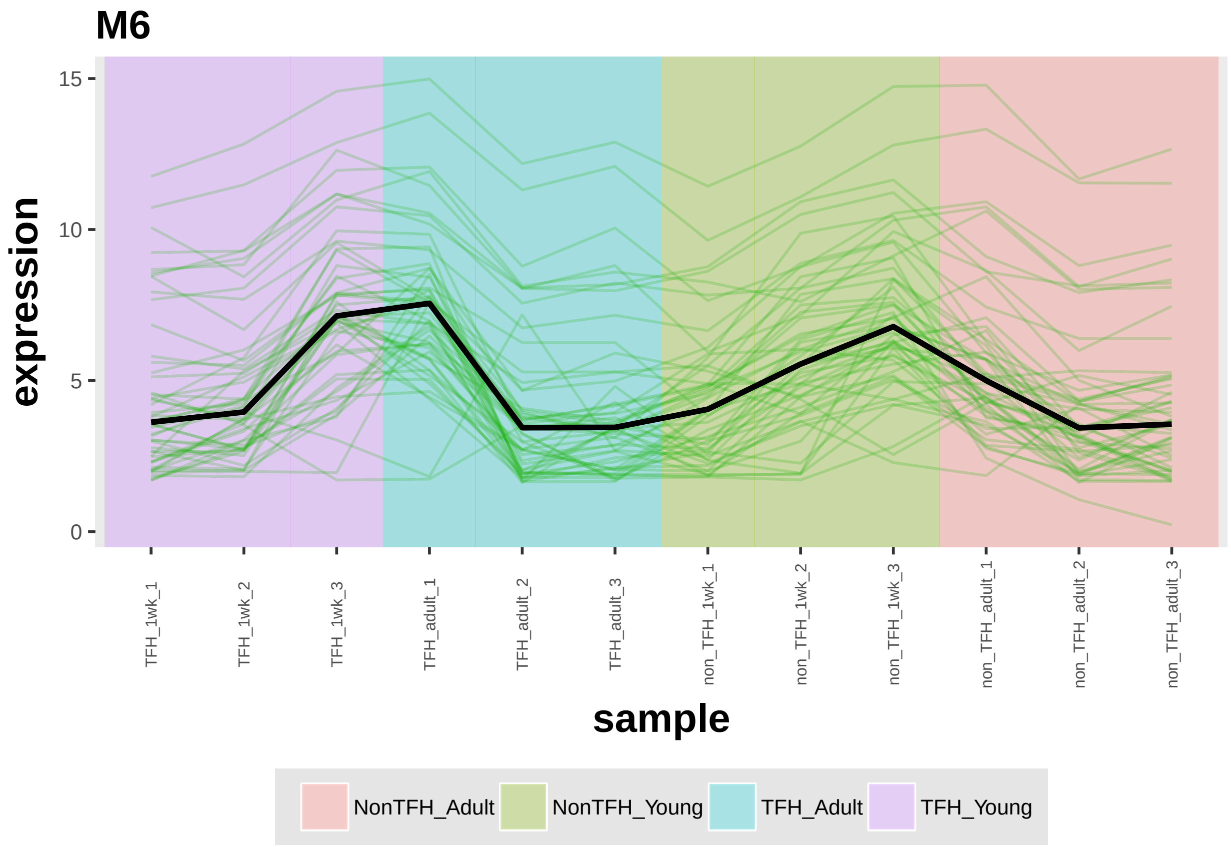

1.3 Gene Set Enrichment Analysis

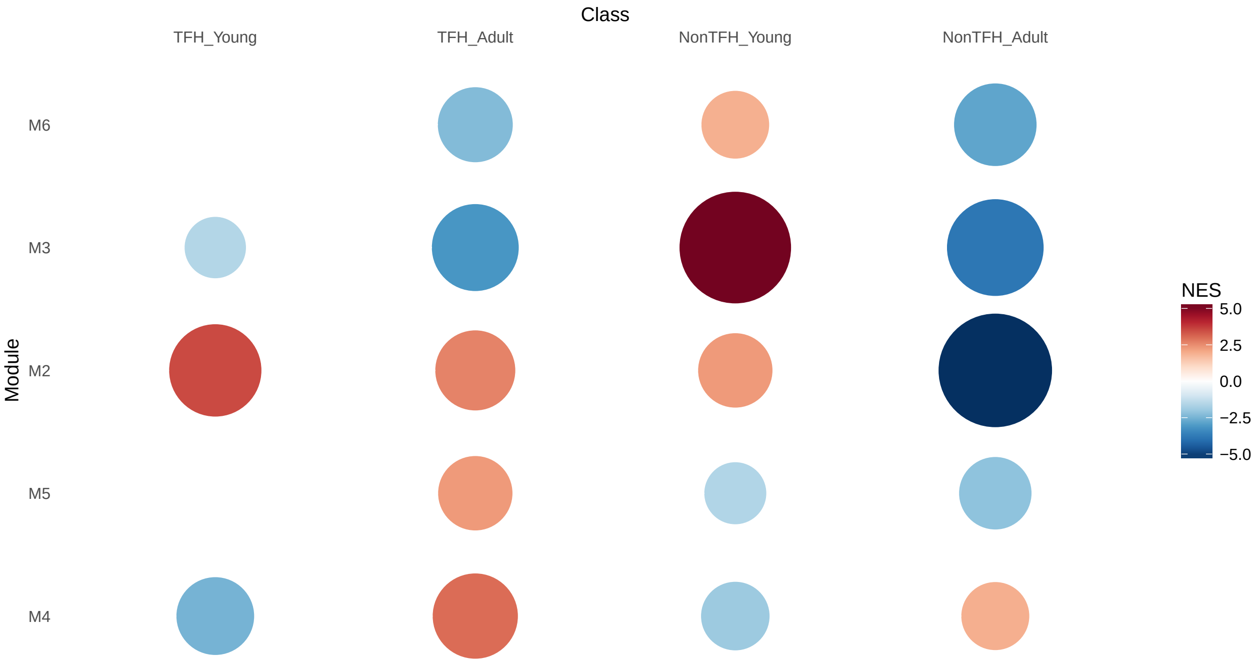

1.4 Over Representation Analysis

1.4.1 M1

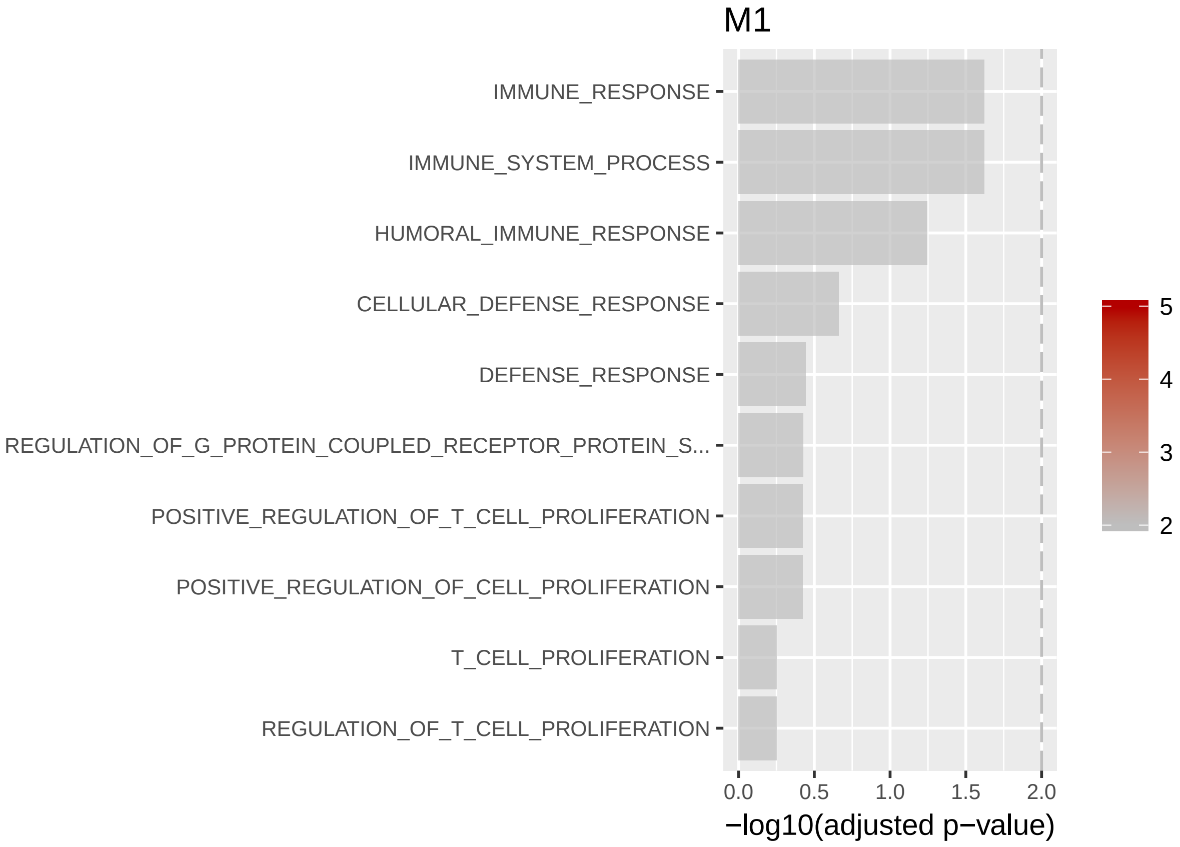

1.4.2 M2

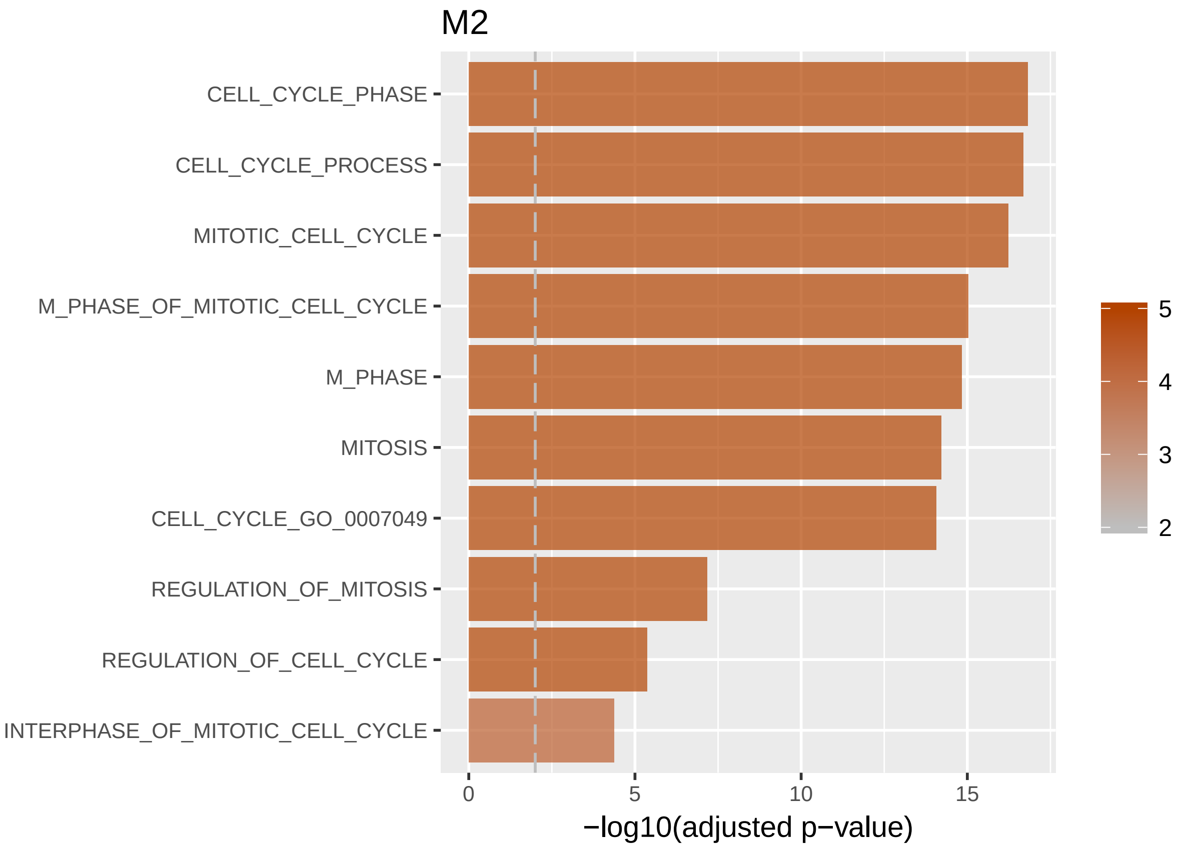

M2
CELL_CYCLE_PHASE
CELL_CYCLE_PROCESS
MITOTIC_CELL_CYCLE
M_PHASE_OF_MITOTIC_CELL_CYCLE
M_PHASE
MITOSIS
CELL_CYCLE_GO_0007049
REGULATION_OF_MITOSIS
REGULATION_OF_CELL_CYCLE
INTERPHASE_OF_MITOTIC_CELL_CYCLE
0
5
10
15
−log10(adjusted p−value)
5
4
3
2

1.4.3 M3

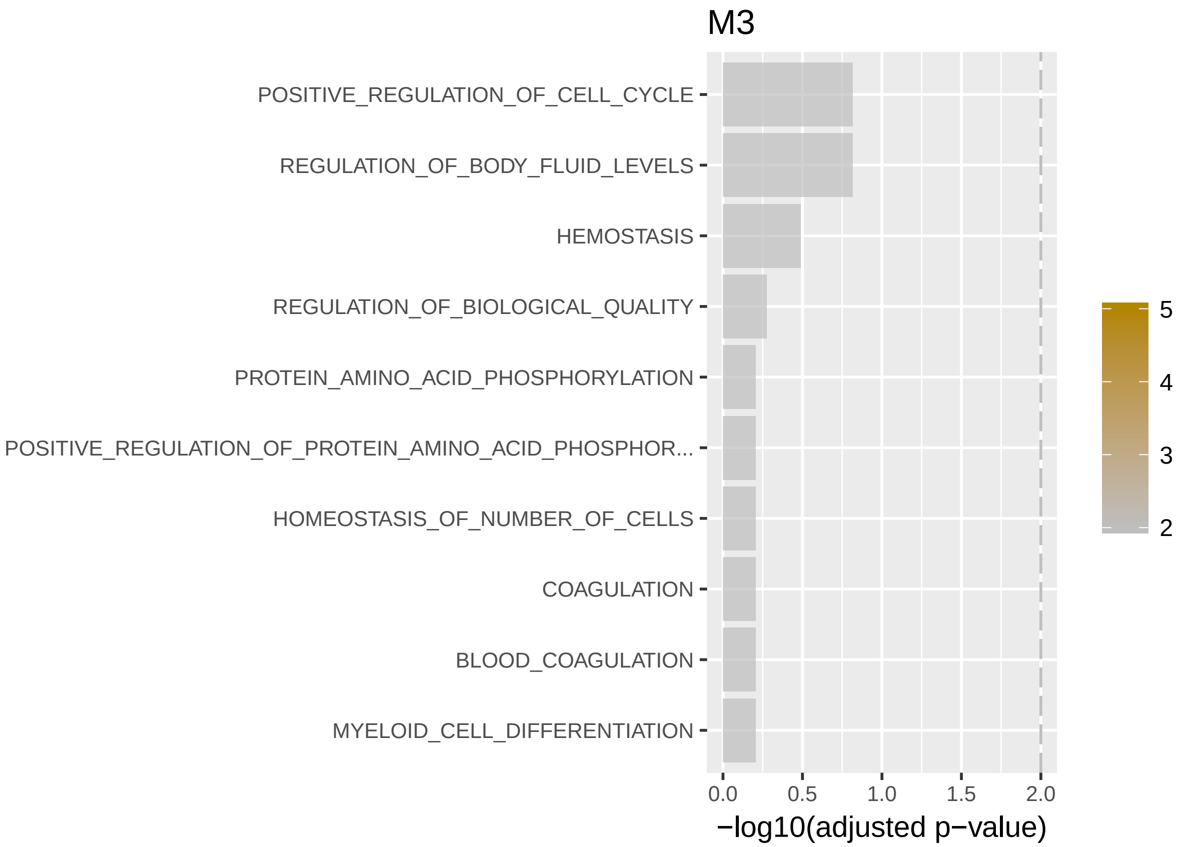
M3
POSITIVE_REGULATION_OF_CELL_CYCLE
REGULATION_OF_BODY_FLUID_LEVELS
HEMOSTASIS
REGULATION_OF_BIOLOGICAL_QUALITY
PROTEIN_AMINO_ACID_PHOSPHORYLATION
POSITIVE_REGULATION_OF_PROTEIN_AMINO_ACID_PHOSPHOR...
HOMEOSTASIS_OF_NUMBER_OF_CELLS
COAGULATION
BLOOD_COAGULATION
MYELOID_CELL_DIFFERENTIATION
0.0
0.5
1.0
1.5
2.0
−log10(adjusted p−value)
5
4
3
2

1.4.4 M4

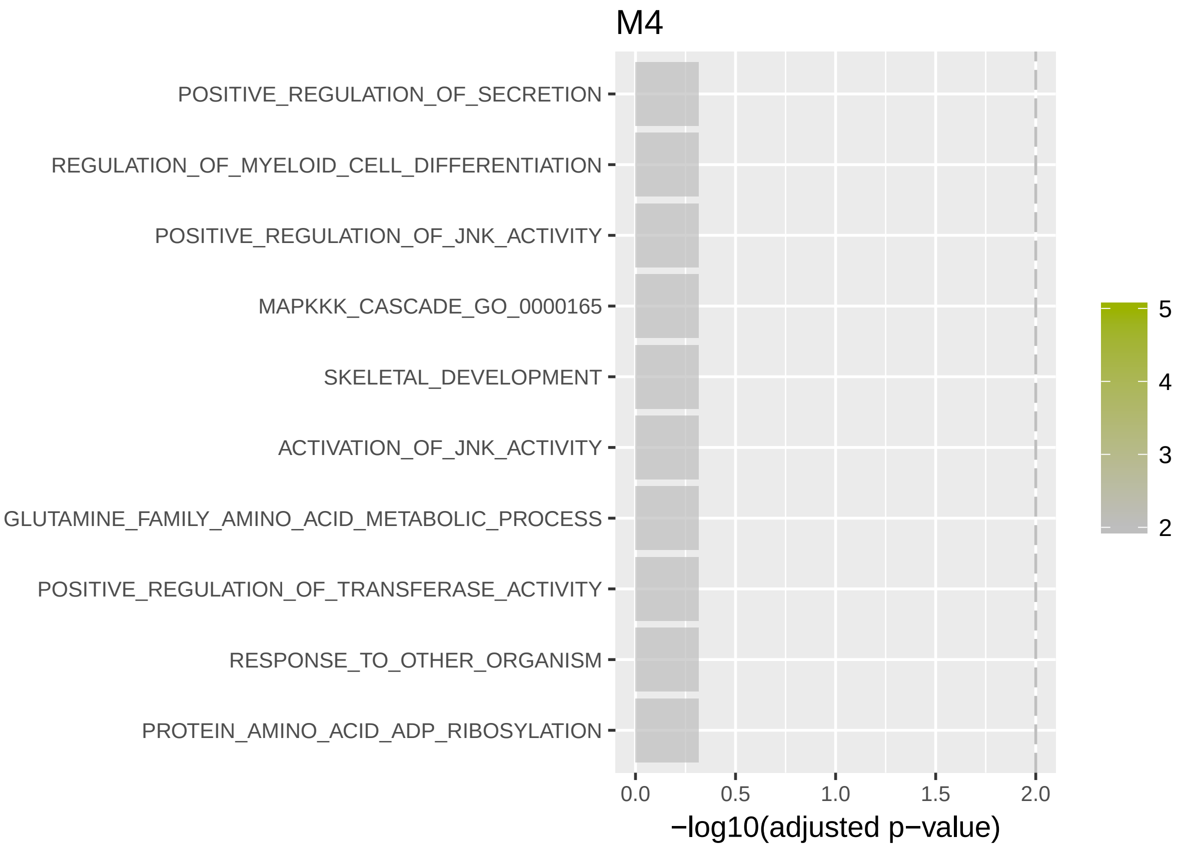
M4
POSITIVE_REGULATION_OF_SECRETION
REGULATION_OF_MYELOID_CELL_DIFFERENTIATION
POSITIVE_REGULATION_OF_JNK_ACTIVITY
MAPKKK_CASCADE_GO_0000165
SKELETAL_DEVELOPMENT
ACTIVATION_OF_JNK_ACTIVITY
GLUTAMINE_FAMILY_AMINO_ACID_METABOLIC_PROCESS
POSITIVE_REGULATION_OF_TRANSFERASE_ACTIVITY
RESPONSE_TO_OTHER_ORGANISM
PROTEIN_AMINO_ACID_ADP_RIBOSYLATION
0.0
0.5
1.0
1.5
2.0
−log10(adjusted p−value)
5
4
3
2

1.4.5 M5

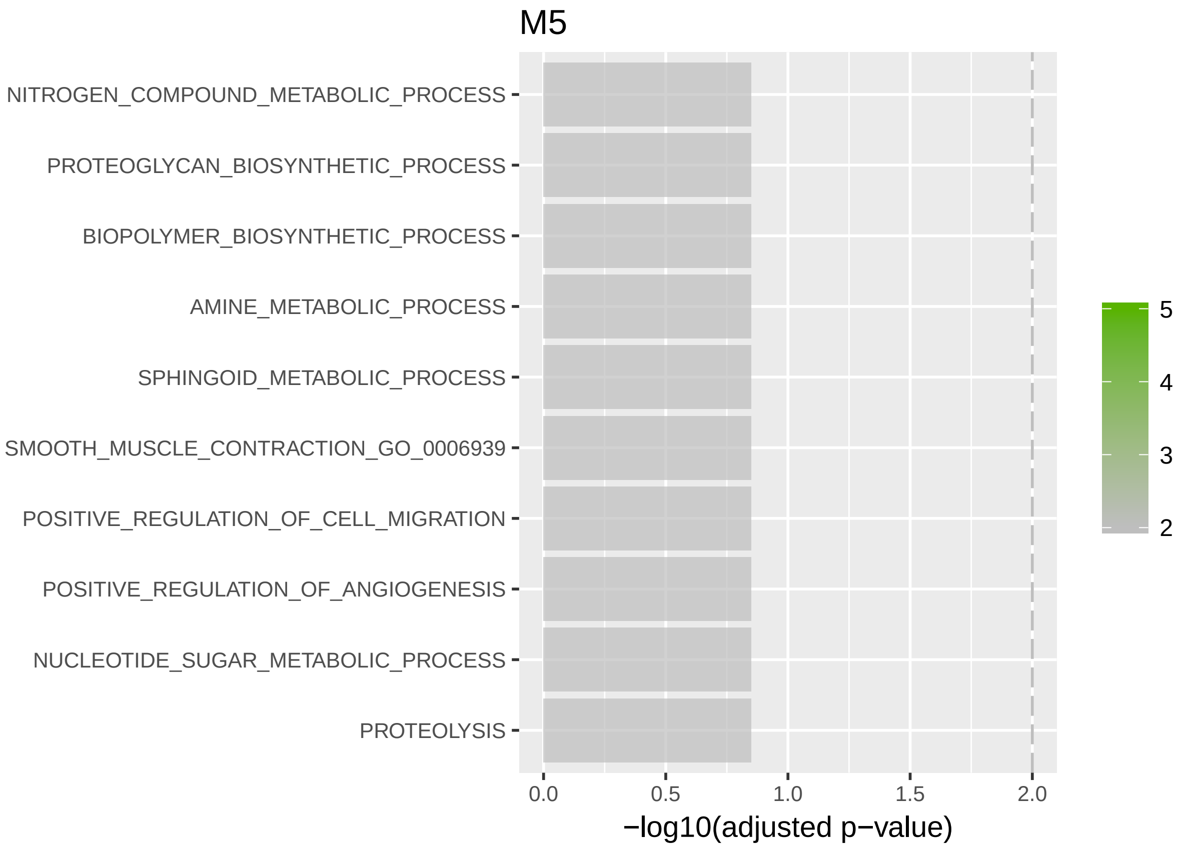
M5
NITROGEN_COMPOUND_METABOLIC_PROCESS
PROTEOGLYCAN_BIOSYNTHETIC_PROCESS
BIOPOLYMER_BIOSYNTHETIC_PROCESS
AMINE_METABOLIC_PROCESS
SPHINGOID_METABOLIC_PROCESS
SMOOTH_MUSCLE_CONTRACTION_GO_0006939
POSITIVE_REGULATION_OF_CELL_MIGRATION
POSITIVE_REGULATION_OF_ANGIOGENESIS
NUCLEOTIDE_SUGAR_METABOLIC_PROCESS
PROTEOLYSIS
0.0
0.5
1.0
1.5
2.0
−log10(adjusted p−value)
5
4
3
2

1.4.6 M6

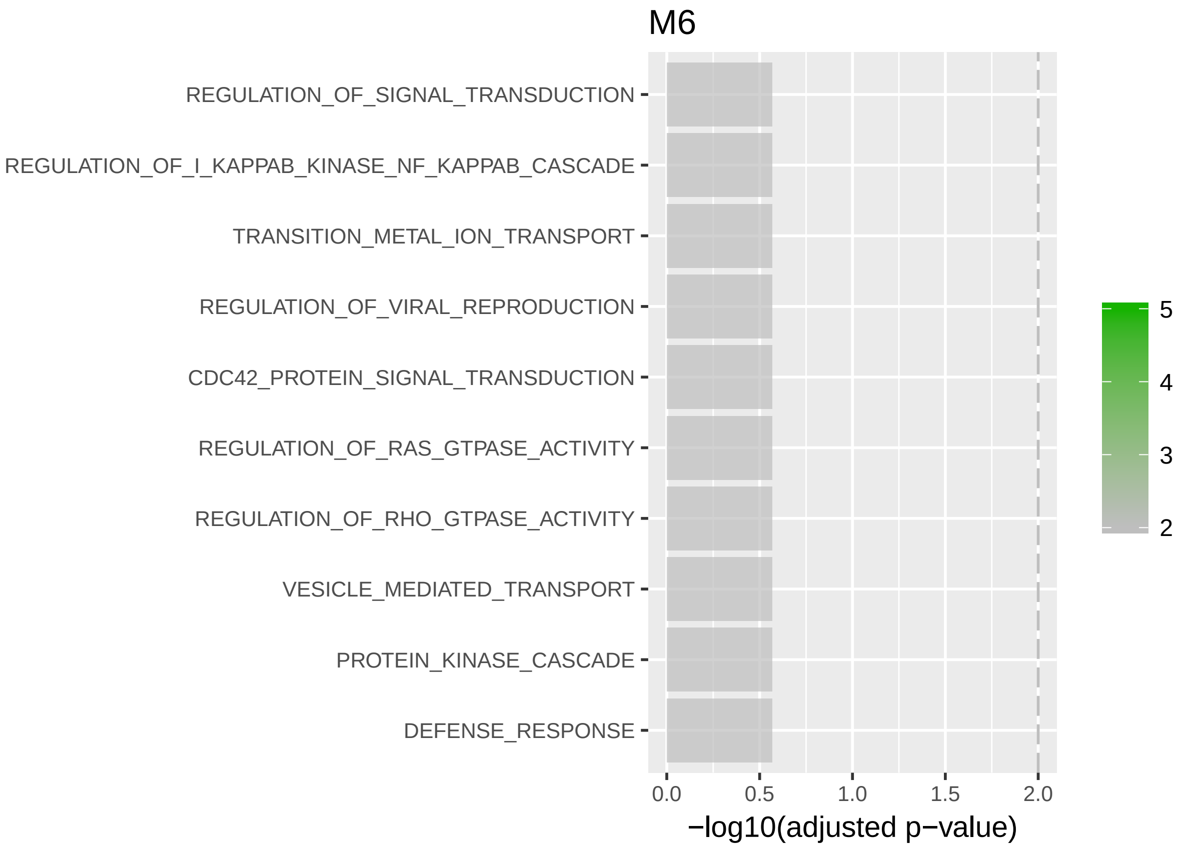
M6
REGULATION_OF_SIGNAL_TRANSDUCTION
REGULATION_OF_I_KAPPAB_KINASE_NF_KAPPAB_CASCADE
TRANSITION_METAL_ION_TRANSPORT
REGULATION_OF_VIRAL_REPRODUCTION
CDC42_PROTEIN_SIGNAL_TRANSDUCTION
REGULATION_OF_RAS_GTPASE_ACTIVITY
REGULATION_OF_RHO_GTPASE_ACTIVITY
VESICLE_MEDIATED_TRANSPORT
PROTEIN_KINASE_CASCADE
DEFENSE_RESPONSE
0.0
0.5
1.0
1.5
2.0
−log10(adjusted p−value)
5
4
3
2

1.5 Interaction Network

M1

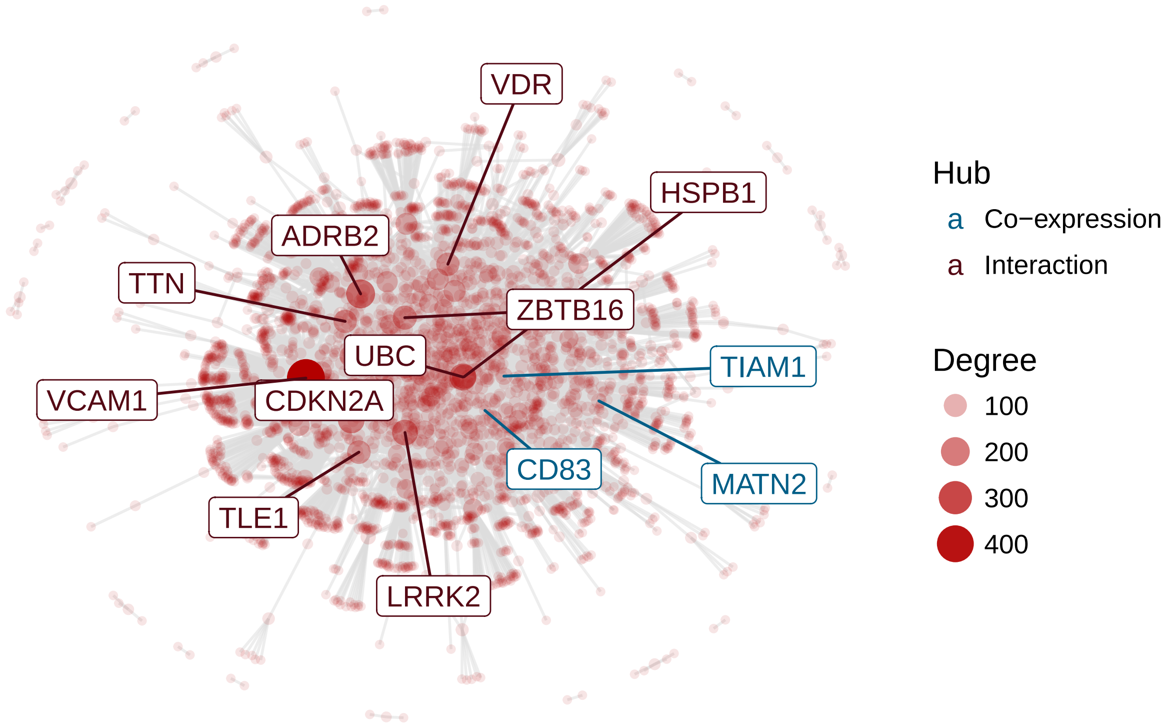

M2

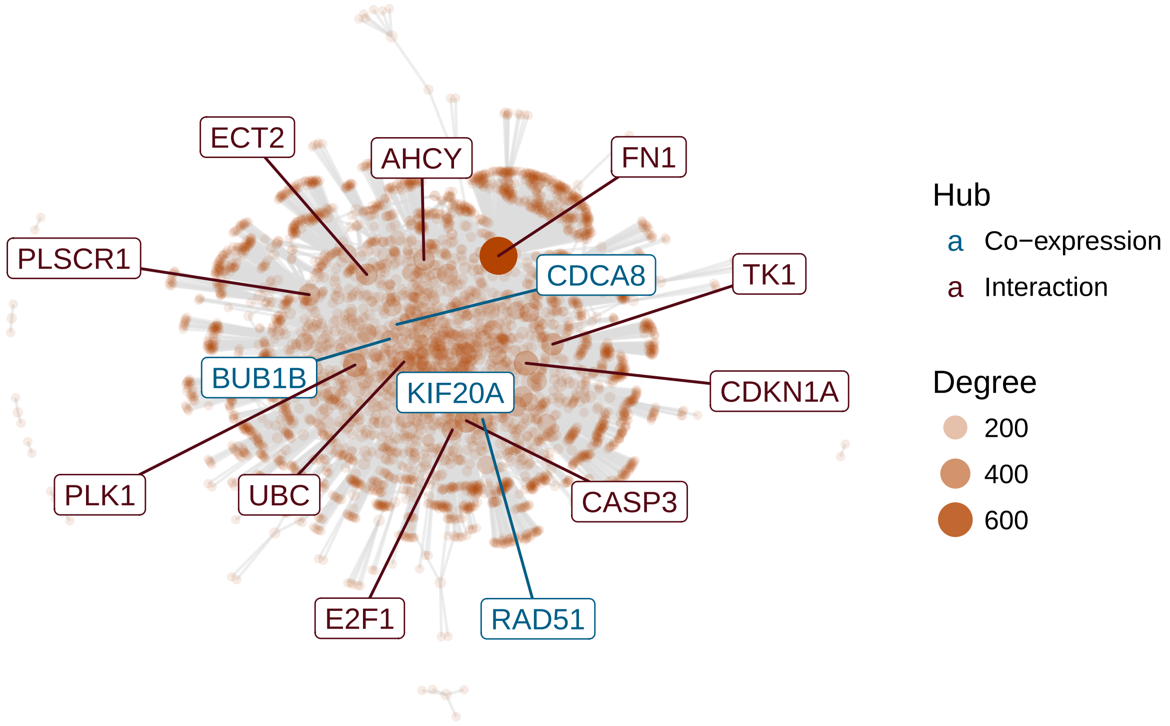

M3

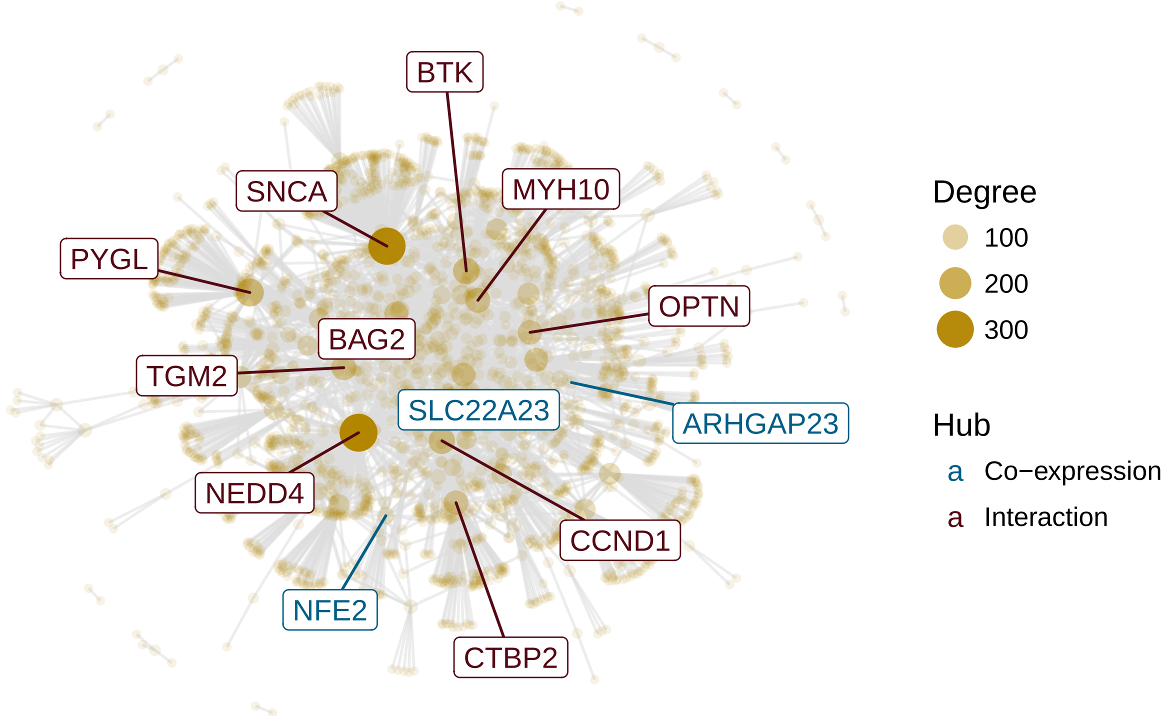

BTK
SNCA
MYH10
PYGL
OPTN
BAG2
TGM2
SLC22A23
ARHGAP23
NEDD4
CCND1
NFE2
CTBP2
Degree
100
200
300
Hub
a Co−expression
a Interaction

M4

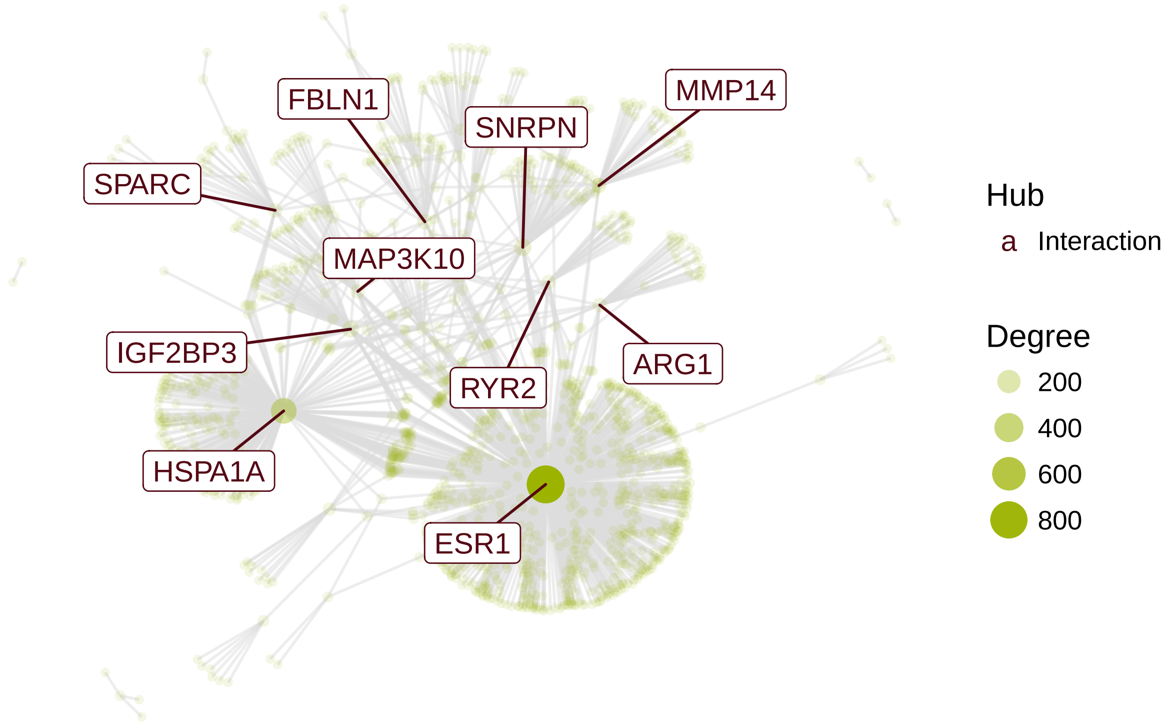

FBLN1
MMP14
SNRPN
SPARC
MAP3K10
IGF2BP3
ARG1
RYR2
HSPA1A
ESR1
Hub
a Interaction
Degree
200
400
600
800

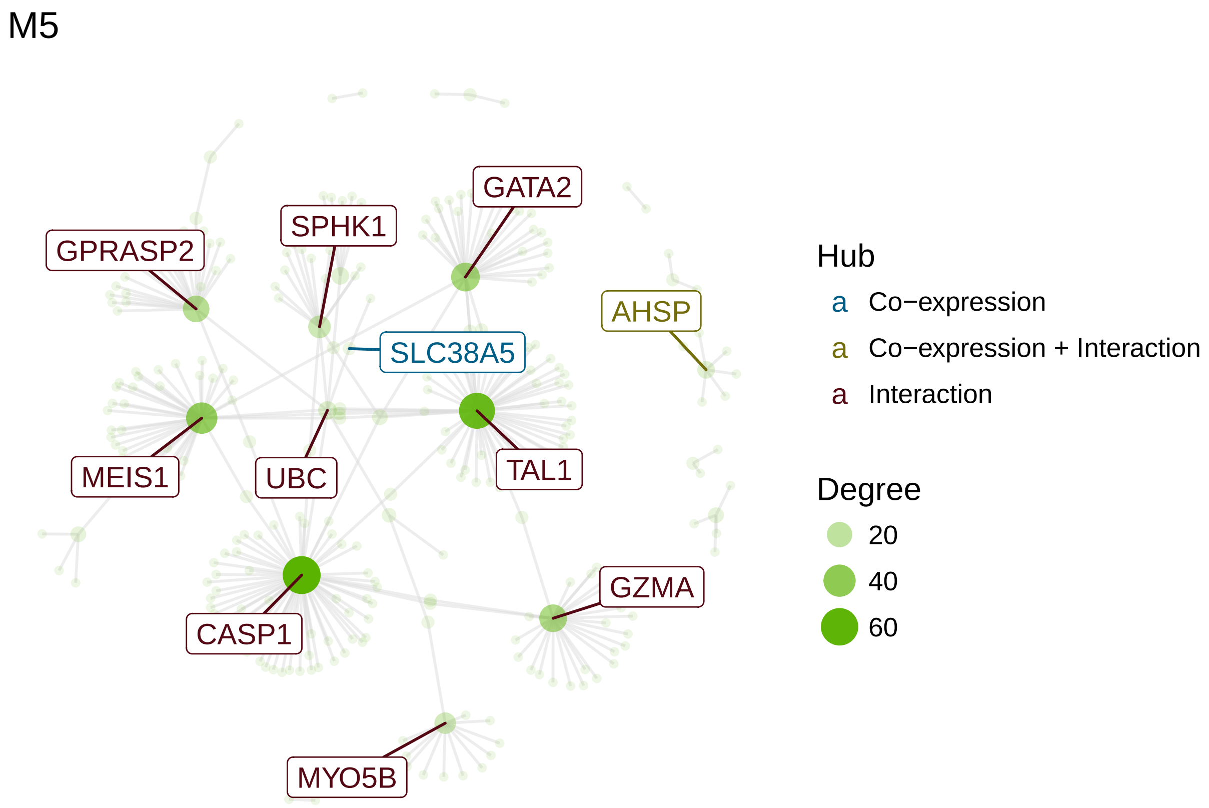

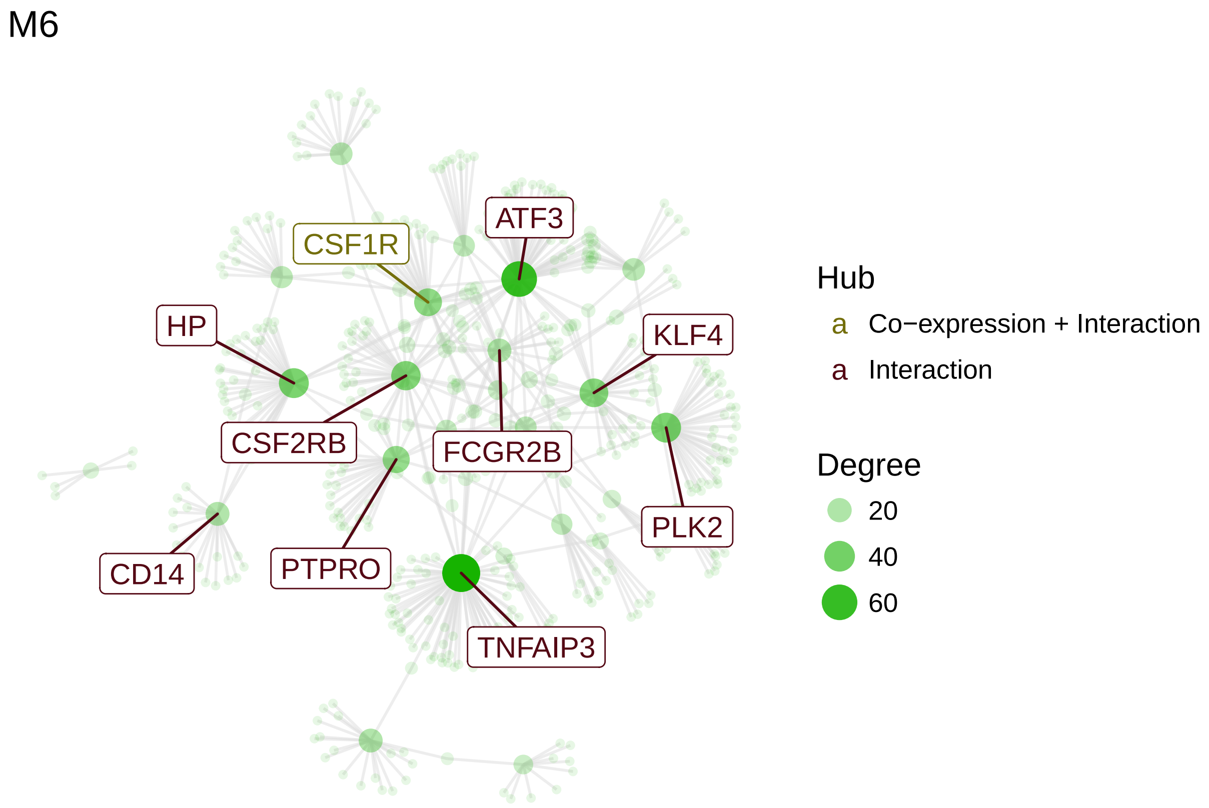

1.6 Parameters
